# Injectable electrospun fiber-hydrogel composite sequentially releasing clonidine and ropivacaine for prolonged and walking regional analgesia

**DOI:** 10.7150/thno.74845

**Published:** 2022-06-21

**Authors:** Sufang Chen, Weifeng Yao, Haixia Wang, Tienan Wang, Xue Xiao, Guoliang Sun, Jing Yang, Yu Guan, Zhen Zhang, Zhengyuan Xia, Mingqiang Li, Yu Tao, Ziqing Hei

**Affiliations:** 1 Department of Anesthesiology and Center for Nanomedicine, The Third Affiliated Hospital, Sun Yat-sen University, Guangzhou 510630, China.; 2 Department of Medicine, The University of Hong Kong, Hong Kong 999077, China.; 3 Laboratory of Biomaterials and Translational Medicine, The Third Affiliated Hospital, Sun Yat-sen University, Guangzhou 510630, China.; 4 Guangdong Provincial Key Laboratory of Liver Disease Research, Guangzhou 510630, China.

**Keywords:** ropivacaine, clonidine, electrospun fiber, injectable hydrogel, long-acting regional analgesia, sensorimotor separation

## Abstract

**Rationale**: Peripheral nerve block is a traditional perioperative analgesic method for its precise pain control and low systemic toxicity. However, a single low dose of local anesthetic merely provides a few hours of analgesia, and high dose results in irreversible toxicity, whereas continuous infusion of anesthetics is expensive and complicated. Therefore, it is necessary to develop a long-acting and sensory-selective local anesthetic for safe perioperative analgesia.

**Methods**: An injectable composite comprising ropivacaine-loaded poly (ε-caprolactone) electrospun fiber and clonidine-loaded F127 hydrogel (Fiber-Rop/Gel-Clo composite) was developed for long-acting and walking regional analgesia with barely one dose. The peripheral nerve blockade effect of the composite was evaluated in a rat sciatic nerve block model. Also, the biodegradability and biosafety of the composite was evaluated.

**Results**: The preferentially released Clo from the hydrogel rapidly constricted the peripheral arterial vessels, reducing the blood absorption of Rop and thus enhancing the local Rop accumulation at the injection site. The subsequently sustainable release of Rop from the fiber, significantly prolonged the sciatic nerve block of rats. Remarkably, an amazing sensorimotor segregation effect was achieved, as the sensory blockade (32.0 ± 1.4 h) lasted significantly longer than the motor blockade (20.3 ± 0.9 h). Additionally, the Fiber-Rop/Gel-Clo composite presented good biodegradability and biosafety *in vivo*.

**Conclusions**: Our designed Fiber-Rop/Gel-Clo composite with minimal invasion, prolonged synergistic analgesia, and strikingly sensorimotor segregation effect, posted a promising prospect for regional long-term walking analgesia in clinical treatment.

## Introduction

Perioperative analgesia is a key procedure for the management and rapid recovery of patients 1, while inefficient pain management may cause a series of complications, including delayed wound healing, prolonged hospital stay, hospital-acquired infection, or even development of chronic pain 2. Recently, peripheral nerve block (PNB) analgesia has been increasingly applied for perioperative analgesia, because it provides exactly walking regional analgesia with marginal systemic toxicity 3. PNB refers to blocking the uploading of pain signals at the regional level, thereby reducing the perception of pain by the central nervous system. Walking regional analgesia combines the rapid pain relief from the regional block, whereas allows sufficient motor function for patients to ambulate 4. Thus, walking regional analgesia are commonly used and very effective for postoperative analgesia in orthopaedic lower limb surgery and intrapartum analgesia. In general, PNB, as a local analgesia method, can be achieved by either a single injection of local anesthetics or continuous infusion with the assistance of drug delivery system. However, a single dose of local anesthetics only provides a few hours of pain relief 5, whereas continuous infusion *via* an indwelling catheter is often inconvenient, complicated, expensive, and frequently associated with complications, such as catheter-associated infections, catheter tip displacement, or nerve injury. Additionally, a facile anesthetic system which provides exact regional pain control without motor blocking is an expectant desire from patients 6. Therefore, it is highly necessary and urgent to develop a facile long-acting and sensory-selective local anesthetic delivery system for effective regional anesthesia and pain management.

The main objective of developing new local anesthetic delivery system is to achieve long-acting effect with selective sensory block and few side effects by a single dose 7. In recent years, some researchers have reported the applications of new local anesthetic agents for prolonged effect. For instance, a bupivacaine-loaded poly (lactic-*co*-glycolic acid) (PLGA) electrospun nanomembrane and a bupivacaine-loaded hydrogel/microsphere composite were reported to significantly prolong the duration time of rat sciatic nerve blockade 8, 9. However, the drug-loaded electrospun nanomembrane was usually administered by incision, and most of the local anesthetic-polymer delivery systems could not achieve selective sensory block. QX-314 (*N*-ethyl-lidocaine), a permanently charged lidocaine derivative, could produce selective local analgesia when administered with capsaicin 10. Nevertheless, QX-314 could induce myotoxicity and neurotoxicity at higher doses 11, 12. The first FDA-approved bupivacaine-loaded liposome (Exparel) is referred to control postoperative pain recently 13, however, the preponderance of evidence fails to support the routine use of liposomal bupivacaine over standard local anesthetics 14.

Ropivacaine (Rop), a frequently used local anesthetic, with relatively long-acting analgesia, presents low cardiotoxicity and sensorimotor separation at low concentrations 15. The duration of single dose lasts less than 6 h, whereas increasing the dose does not prolong the anesthetic effect, instead, severe neurotoxic and cardiotoxic effects appeared. The addition of adjuvants has been confirmed to be a potential method for prolonging the blockade effect of local anesthetics 16. Clonidine (Clo) is a centrally acting antihypertensive agent with α2 adrenoceptor agonist properties. Recently, Clo has been used to extend the duration of local anesthetics in some clinical studies, showing favorable effects 17, 18, but the exact mechanism is unclear yet.

Currently, nanomedicine and preclinical investigations are gaining increasing attention in pharmaceutical and biomedical research 19-22, among which the injectable, thermo-sensitive hydrogel has been widely used in drug delivery system 23. A series of thermosensitive polymer hydrogels, such as poly (ethylene glycol)-block-poly (ε-caprolactone)-graft-poly (2-(guanidyl) ethyl methacrylate) (mPEG-*b*-PCL-*g*-PGEM, PECG), poly (DL-lactide-*co*-glycolide-*b*-ethylene glycol-*b*-DL-lactide-*co*-glycolide, PLGA-PEG-PLGA) and chitosan hydrogels 24-26, have been applied as delivery systems for local anesthetics. Electrospun fiber is a new form of polymer morphology that can be easily produced with an electrospinning device and different polymer solutions. With high flexibility and drug loading efficiency, the electrospun fiber could prevent the burst release and enable a sustained drug release for longer periods 27. These advantages endow the cargo-loaded fibers with wide applications in anti-bacterial, hemostatic dressing, anti-tumor, and tissue engineering fields, *etc* 28.

**Scheme 1:**
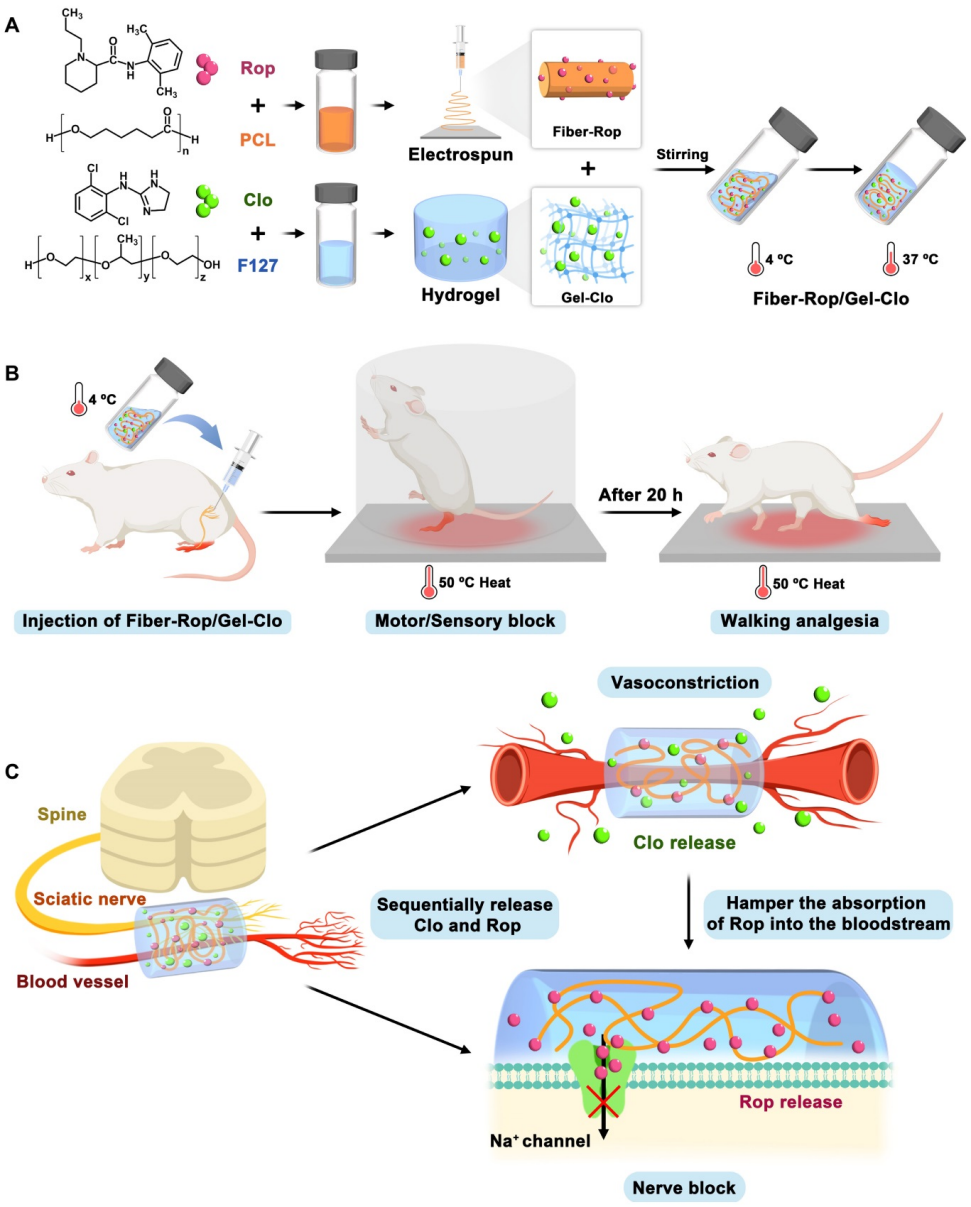
Schematic illustration of fabrication and sciatic nerve blockade effect of the Fiber-Rop/Gel-Clo composite. (A) The Fiber-Rop/Gel-Clo composite was fabricated by combining Rop-loaded electrospun PCL nanofiber and Clo-loaded F127 hydrogel. (B) The rat sciatic nerve blockade effect of the Fiber-Rop/Gel-Clo composite. (C) The mechanism of long-acting and walking regional analgesia *in vivo*. Created with BioRender.com.

Herein, we constructed an injectable, regional anesthetic composite by combining Rop-loaded electrospun nanofiber and Clo-loaded F127 hydrogel (Fiber-Rop/Gel-Clo composite) for prolonged and sensory-selective regional analgesia with only a single injection. The preferentially released Clo from the hydrogel rapidly constricted the peripheral arterial vessels, dramatically hampering the blood absorption of Rop and thus effectively enhancing the local Rop accumulation at the injection site. The steadily released Rop from the electrospun fiber substantially blocked the Na^+^ channel and prolonged the local sciatic nerve block of rats (**Scheme 1**). It is worth noting that the sensory blockade (32.0 ± 1.4 h) lasted significantly longer than the motor blockade (20.3 ± 0.9 h), indicating the remarkable sensorimotor segregation effect of the composite. In addition, the Fiber-Rop/Gel-Clo composite presented good biodegradability and satisfactory biosafety* in vivo*. Therefore, the fabricated Fiber-Rop/Gel-Clo composite, which possesses smooth injectability, minimal invasion effect, prolonged regional analgesia, and remarkable sensorimotor segregation effect, may provide a potential strategy for perioperative regional anesthesia and pain management.

## Materials and Methods

### Materials

Poly(ε-caprolactone) (PCL, [η] = 1.8 ± 0.2 dL/g) was purchased from Polymtek (Shenzhen, China). Pluronic® F127 (F127, molecular weight) was obtained from Sigma-Aldrich (St. Louis, MO, USA). The ropivacaine base (Rop), clonidine hydrochloride (Clo), and lipase were purchased from Macklin (Shanghai, China). Ropivacaine hydrochloride solution (Rop-HCl) was obtained from AstraZeneca AB (SE-151 85 Sodertalje, Sweden). Fluorescein isothiocyanate (FITC), Rhodamine B (RhB), dichloromethane (DCM), methanol, and acetone were purchased from Aladdin (Shanghai, China). Toluidine blue O (TBO) dye, TUNEL apoptosis assay kit, diaminobenzidine (DAB), TNF-α, and IL-6 rabbit pAb were purchased from Servicebio (Wuhan, China).

### Fabrication of PCL-Rop electrospun fiber

PCL (20%, wt%) and Rop (60%, wt%) were dissolved in DCM, and the solution was stirred with a magnetic stir bar (MYP84-1, Shanghai, China) for 2 h at room temperature. The solution was injected with a 25-G stainless steel needle at a controlled rate of 1.08 mL/h, and steady spiral-like fibers were formed under a 9 kV electrostatic field. The distance between the needle and the rotating collector (running at 400 rpm) was 10 cm. All electrospun fibers were fabricated at a room temperature of 24 ± 0.5 °C and humidity of 40 ± 5%. The electrospun fiber membrane was stored in a vacuum drying oven to further remove the organic solvent.

For the determination of the drug loading content (DLC) and the drug loading efficiency (DLE) of Rop, the absorbance at 263 nm was extracted from the detected absorption spectra upon dissolving dried Fiber-Rop with methanol. The DLC and DLE of Rop were calculated as: DLC (%) = (mass of loaded drug)/(total mass of fibers) × 100%, DLE (%) = (mass of loaded drug)/(initial mass of drug) × 100%.

### Fabrication of F127 hydrogel

F127 solutions with a series of different concentrations (20%, 25%, 30%, 35%, and 40%; wt%) were prepared by dissolving Pluronic® F127 into sterile Milli-Q water. The solution was stirred manually for 20 min to fully dissolve F127, which was subsequently frozen for 5 min at -80 °C and stored in a 4 °C refrigerator. The 40% F127 solution was chosen to produce a thermo-sensitive and injectable hydrogel.

### Fabrication of Fiber-Rop/Gel-Clo composite

By adding Fiber-Rop and Clo into a 4 °C F127 solution (40%, wt%), the mixture solution was vortexed to mix thoroughly. The mass ratio of Rop and Clo in this composite was 2000:1 as previously described 29. Afterwards, 26.7 mg Fiber-Rop (containing 20 mg Rop) and 10 μg Clo were added to 1 mL F127 solution and vortexed at 4 °C for 1 h to obtain a homogeneous suspension. Then, the suspension was kept in a thermostatic water bath (Shanghai Scientific Instrument Co., Ltd., China) at 37 °C for 10 min to obtain the Fiber-Rop/Gel-Clo composite.

### Characterization of Fiber-Rop/Gel-Clo composite

The surface morphology of the freeze-dried PCL fibers (Rop-loaded or unloaded), F127 hydrogel, and Fiber-Rop/Gel-Clo composite was examined *via* scanning electron microscopy (SEM; JSM-6330F, JEOL, Japan) with an acceleration voltage of 20 kV. The average fiber diameter was then calculated by measuring 100 fibers from five different fields of each electrospun mat. In addition, Fiber-Rop and Gel-Clo were labeled by FITC and RhB, respectively, and the distribution of labeled fiber and F127 hydrogel in the Fiber/Gel composite was characterized by fluorescence microscope (Ni-U, Nikon, Japan). Briefly, for the preparation of FITC-labeled fiber (Fiber-Rop-FITC), FITC (1%, wt%) was added into the solution comprising PCL (20%, wt%) and Rop (60%, wt%) to generate the electrospun fiber. To mark F127 hydrogel with RhB (Gel-Clo-RhB), RhB (1%, wt%) was homogenously mixed with F127-Clo solution at 4 °C. Then the composite of Fiber-Rop-FITC/Gel-Clo-RhB was obtained as described above. Finally, the Fiber-Rop-FITC/Gel-Clo-RhB composite was gently spread on the surface of a clean microscope slide before being observed by a fluorescent microscope.

The thermosensitivity, *i.e.* sol-gel transition property, of the F127 hydrogel and Fiber-Rop/Gel-Clo composite was determined according to rheological behavior. The rheological properties, storage modulus (G′), and loss modulus (G″) of the F127 hydrogel and Fiber/Gel composite were recorded at 1% strain and 1.0 Hz frequency with the temperature ranging from 0 °C to 40 °C at a speed of 2 °C/min *via* a HAAKE MARS 60 dynamic shear rheometer (Thermo Scientific, USA).

### In vitro drug release of Fiber-Rop/Gel-Clo composite

The drug release test was scheduled for 14 days. 1 mL Fiber-Rop/Gel (containing 10 mg Rop) or Fiber/Gel-Clo (containing 1 mg Clo) composite solution was transferred into a dialysis bag (MWCO, 3500 Da, Sigma-Aldrich, USA) and then put into a 20 mL vial. The vial was kept at 37 °C for 10 min to form the Fiber/Gel composite. Two groups (n = 3 of each) of vials containing composites to be dialyzed were added slowly with phosphate-buffered saline (PBS) solution with or without 20 U/mL lipase, which was subsequently incubated in a 37 °C shaking incubator at a speed of 40 rpm. At predetermined time points (0, 10 min, 30 min, 1 h, 1.5 h, 2 h, 3 h, 4 h, 5 h, 6 h, 7 h, 8 h, 9 h,10 h, 24 h, 36 h, 2 day, 3 day, 4 day, 5 day, 6 day, 7 day, 9 day, 12 day, and 14 day), 300 μL dialyzed solution was withdrawn and replaced with 300 μL fresh PBS solution. The concentrations of Rop and Clo released into the buffered solution were detected by UV-Vis spectrophotometer at 263 nm and 270 nm, respectively. Standard Rop and Clo solutions were used to generate linear calibration curves (R^2^ > 0.99, **Figure S1**), which were subsequently applied for determining the concentrations of Rop and Clo in all tested samples.

### In vitro degradation of composite

*In vitro* degradation of the Fiber/Rop was assessed by photographs captured by a digital camera and weighing method. The initial morphology of Fiber/Rop was snapped and the weight (W_0_) with equal mass was recorded after lyophilizing for 24 h. Thereafter, 15 mL PBS solution without or with 20 U/mL lipase was added to the vials containing Fiber/Rop, and then these vials were incubated in a 37 °C shaking incubator at a speed of 40 rpm. The solution in the vial was withdrawn and replaced with the same kind of solution every three days. At predetermined time points (0 day, 3 day, 6 day, 9 day, 12 day, 15 day, 18 day, and 21 day), the remaining weights (W_1_) of the composite were recorded. The latter was calculated as follows: remaining weight (%) = W_1_/W_0_ × 100%. In addition, to explore the diffusion of the F127 gel *in vitro*, 1% RhB was added to the gel for better visual observation. At predetermined time points (0 h, 0.5 h, 1 h, 2 h, 4 h, 6 h, 12 h, and 24 h), the morphology of the Gel-RhB was recorded by a digital camera.

### Rat model of sciatic nerve block

All animal-related processes gained approval from the Animal Care Committee of South China Agricultural University (No. 2021d037). Adult male 250-300 g Sprague-Dawley (SD) rats were purchased from Vital River Laboratory Animal Technology Co., Ltd. (Zhejiang, China) and housed in ventilated cages in a 12-h light-dark cycle (8:00-20:00 light; 20:00-8:00 dark). All rats were acclimated to experimental environments for 1 week with free access to food and water, before being randomly divided into seven groups (6 rats/group): Saline (0.9% saline), Rop (5 mg Rop hydrochloride in PBS), Clo (2.5 μg Clo hydrochloride in PBS), Rop + Clo (5 mg Rop hydrochloride and 2.5 μg Clo hydrochloride in PBS), Fiber/Gel (blank Fiber/Gel composite), Fiber-Rop/Gel (with 20 mg Rop), and Fiber-Rop/Gel-Clo (with 20 mg Rop and 10 μg Clo). Under inhalation anesthesia with 2.0% sevoflurane (Hengrui Pharmaceuticals Co., Ltd, Jiangsu, China), each group of rats received left peri-sciatic nerve injections with 1 mL of the corresponding solution (4 °C) as described in the seven groups *via* a 1.2 mm needle.

### Evaluation of peripheral nerve blockade

Baseline values of the sensory and motor block tests were recorded daily for three days before injection of our examined materials. Neurobehavioral tests were performed every hour in the first 6 h, then the interval was changed to every 2 h for the rest of the experiment. Motor block of the limb with injection was evaluated with a four-point rating scale (**Video S1**): 1, no motor block; 2, dorsiflexion disorder and failure to fully splay the toes when lifting the rat's tail; 3, plantarflexion disorder and complete failure to splay the toes when lifting the rat's tail; 4, complete loss of dorsiflexion and plantarflexion accompanied by gait disorders. Duration of motor block was the time from score 4 to score 2.

The hot plate test was performed to assess the sensory blockade 30 (**Video S2**). Briefly, rats were placed on a hot plate analgesia tester (BME-480, Yuyan, Shanghai, China) at 50 °C ± 0.5 °C with a transparent barrier to avoid rat escape. The time from the onset of the thermal stimulus resulting in licking, withdrawing paw, or jumping was recorded as a rat's paw withdrawal thermal latency (PWTL). A cut-off time was set as 30 s to avoid hyperalgesia and skin damage. Upon reaching 30 s, the rat was removed from the hot plate, and the PWTL was recorded as the maximum value, *i.e.* 30 s. Each heat stimulation was repeated thrice with an interval of 5 min between stimuli, and the mean PWTL was calculated. Percentage of maximum possible effect (% MPE) was calculated as the effect of sensory blockade: %MPE = (PWTL_1_-PWTL_0_) / (Cut-off time-PWTL_0_) × 100% 24. In this equation, PWTL_1_ refers to the PWTL after injection, PWTL_0_ refers to the baseline PWTL, with a cut-off time of 30 s. Effective sensory block was obtained as % MPE reaching 50% and duration of sensory block was the time from % MPE reaching 50% to less than 50%.

### Local vasoconstriction effect of Clo by ultrasound examination

Six SD rats were randomly divided into two groups (n = 3 of each), Fiber-Rop/Gel and Fiber-Rop/Gel-Clo groups. Under inhalation anesthesia with 2.0% sevoflurane, rats were injected with 1 mL Fiber-Rop/Gel composite with or without 10 μg Clo, and subsequently placed in a lateral position. A high-frequency ultrasound (TYPE 1202 B-K Medical ApS, Miieparken 34, DK-2370 Herlev, Denmark) with a 15 Hz linear array probe was used to detect the arterial diameter and blood flow rate around the sciatic nerve at 0 h, 4 h, and 8 h after injection of our examined composite. During ultrasonography, the arterial blood flow was monitored by continuous-wave Doppler mode. The angle between the ultrasonic beam and the direction of blood flow was set at 30°- 60°.

### Effect of Clo on the pharmacokinetics of Rop mimicked by RhB in vivo

For the preparation of RhB-loaded fiber (Fiber-Rop-RhB), RhB (1%, wt%) was added into the PCL (20%, wt%) and Rop (60%, wt%) solution. Fiber-Rop-RhB was obtained as described above. Then 1 mL Fiber-Rop-RhB/Gel composite with or without 10 μg Clo was injected around the sciatic nerve of the rats (n = 3 of each group). At 0 h, 0.5 h, 2 h, 4 h, 6 h, 8 h, 10 h, 12 h, 24 h, 48 h, 72 h, and 96 h after injection, 300 μL blood samples were collected from the tail vein with heparinized centrifuge tubes, and stored overnight at 4 °C. The blood samples were centrifuged at 3000 rpm for 10 min at 4 °C to extract the plasma. After extraction with acetone, the concentrations of RhB in the above samples at different predetermined time points were calculated according to the absorbance at 554 nm from absorption spectra.

### Assessment of local neurotoxicity and inflammation

The rats were euthanized on the 7th postoperative day, sciatic nerves and surrounding muscles were collected for H&E staining, TBO staining, TUNEL assay, and immunohistochemical analysis.

For nerve TBO staining, the sciatic nerve was fixed with 4% paraformaldehyde for 24 h, followed by incubation with 1% osmium for 2 h. Then the samples were dehydrated with gradient ethanol series of 100%, 95%, 85%, and 75%, embedded with epoxy resin, sectioned transversely with 10 μm thickness, stained with 0.1% TBO, and observed under an optical microscope (NSS-6, Nanjing Jiangnan Novel Optics Co., Ltd., China).

TUNEL staining was performed following the manufacturer's instructions. After being deparaffinized, rehydrated, and high-pressure antigen retrieval, paraffin-embedded nerve tissue slides were pretreated with proteinase K solution at 37 °C for 30 min. Next, peroxidase blocking was done by incubation the slides with 3% hydrogen peroxide (H_2_O_2_) for 10 min, which were then incubated in TdT enzyme reaction solution at 37 °C for 60 min. After rinsing with PBS, the slides were incubated with TMR (red) Tunel Cell Apoptosis Detection Kit (G1502-50T) at 37 °C for another 60 min. In addition, the nuclei were stained with DAPI. The degree of neuronal apoptosis was viewed with a fluorescent microscope (Ni-U).

Immunohistochemical analysis of TNF-α and IL-6 was carried out to evaluate the potential inflammation of the sciatic nerves and surrounding muscles. Briefly, after high-pressure antigen retrieval, the slides were rinsed with PBS, and then blocked with 3% H_2_O_2_ for 20 min and 5% goat serum for 30 min at room temperature in order. Then 50 μL rabbit antibody solutions of TNF-α and IL-6 (1: 400) were added to the surface of the sections for incubation overnight at 4 °C. The next day, the slides were rinsed with PBS and incubated with anti-rabbit secondary antibody solution (1: 200) for 60 min at room temperature. Afterwards, the slides were rinsed with PBS, which was added with 50 μL diaminobenzidine (DAB) (1: 50) onto the surface of the sections. The reaction was terminated with distilled water based on the degree of staining. Additionally, the nuclei were stained with hematoxylin. The degree of inflammation was viewed with an optical microscope.

### In vivo degradation of Fiber/Gel composite

To explore the degradation of electrospun Fiber/Gel composite *in vivo*, the injection site was dissected at 7 day, 14 day, and 21 day after injection, to check whether the composite degraded or not.

### Assessment of systemic toxicity

To further evaluate the systemic toxicity caused by the composite, we recorded the mental state and weight change of all rats immediately after sciatic nerve block. On the 7th day post-injection, rats were euthanized for further investigation. Briefly, blood samples collected from the abdominal aorta of live rats were used to detect the levels of serum creatine kinase isoenzyme (CK-MB), lactate dehydrogenase (LDH), aspartate aminotransferase (AST), alanine transaminase (ALT), blood urea nitrogen (BUN) and creatinine (Cr). Then the rats were euthanized with excessive sevoflurane and intracardially perfused with 300 mL 0.9% saline. The heart, liver, spleen, lung, kidney, and brain were collected and fixed with 4% paraformaldehyde, embedded in paraffin, sectioned with 10 μm thickness, stained with hematoxylin/eosin, and observed under the optical microscope.

### Statistical analysis

The statistical results were presented by mean ± standard error (SE). Differences between groups were analyzed by using one-way ANOVA followed by Bonferroni's post hoc test.

## Results

### Fabrication and characterization of Fiber-Rop/Gel-Clo composite

**Figure 1:**
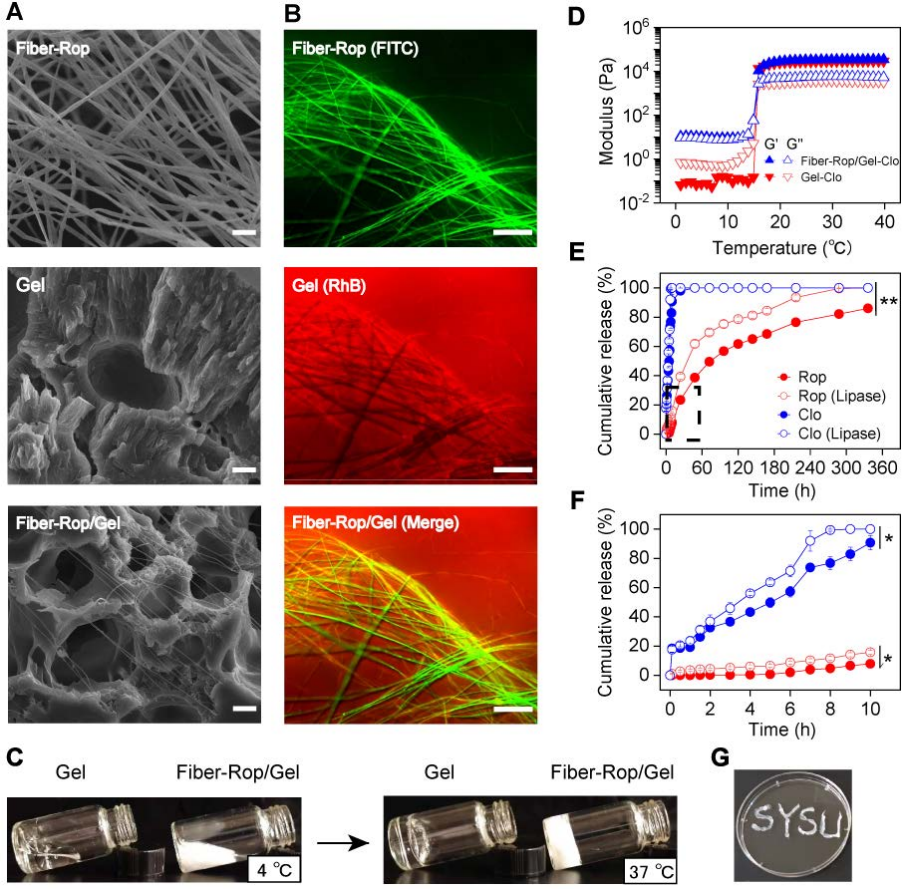
**Characterization of Fiber-Rop/Gel-Clo composite.** (**A**) Scanning electron microscopy (SEM) images of the Rop-loaded fiber (Fiber-Rop), F127 hydrogel (Gel), and Fiber-Rop/Gel composite; Scale bar, 10 µm. (**B**) Fluorescence microscope of fluorescently labeled Rop-loaded fiber (FITC, green), Clo-loaded F127 hydrogel (RhB, red), and Fiber-Rop/Gel-Clo composite (Merge); scale bar, 100 µm. (**C**) Photographs showing F127 hydrogel and Fiber-Rop/Gel composite at 4 °C and 37 °C. (**D**) Rheological behaviors of the Clo-loaded F127 hydrogel and Fiber-Rop/Gel-Clo composite, measured at 1% strain and 1.0 Hz frequency with the temperature set from 0 °C to 40 °C at a speed of 2 °C/min. G′, storage modulus; G″, loss modulus. (**E, F**) *In vitro* cumulative release of Rop and Clo from the Fiber/Gel composite in phosphate buffered saline with or without lipase (20 U/mL), (**E**) 0-14 days, (**F**) 0-10 hours. Data are shown as mean ± standard error, (n = 3/group; *P < 0.05, **P < 0.01). (**G**) Photographs showing the Fiber-Rop/Gel-Clo composite with good injectability.

To acquire an optimum electrospinning-fiber formula, a series of electrospinning parameters have been thoroughly tested on different biodegradable polymer materials, such as polylactic acid (PLA), PLGA, PCL, *etc*. (**Table S1**). Upon altering and optimizing the weight ratio of PCL and Rop, and electrospinning parameters, such as solution injection speed, voltage, solvent, *etc*., a stable cotton-like Rop-loaded PCL fiber (20% PCL, 60% Rop, wt%) was obtained. The drug loading content (DLC) and drug loading efficiency (DLE) of Rop were measured and calculated as 72.4 ± 0.4% and 96.5 ± 0.6%, respectively (**Table S2**), consistent with the formula of the initially prepared PCL and Rop considering of the inevitable drug loss (3.5%) during electrospun fiber fabrication. The quantities of loaded Rop and Clo were determined as 0.72 mg/mg fiber and 10 µg/mL F127 solution by UV absorption spectra, respectively. Secondly, to achieve a minimally invasive drug delivery gel system with great injectability, 40% F127 hydrogel (wt%), was preferred after exploring F127 solutions with different mass fractions (**Table S3**). Upon thoroughly immersing 26.7 mg Fiber-Rop and 10 μg Clo into 1 mL F127 solution at 4 °C, a Fiber-Rop/Gel-Clo gel composite was acquired under 37 °C for 10 min, which could smoothly pass through the injection needle with a diameter of 1.2 mm (**Figure 1G, Figure S2 and Video S3**) and consequently was promoted to be a potential candidate in the minimally invasive delivery system.

Among fabrication of the injectable dual-drug-loaded fiber/gel composite, the fiber phase, gel phase, and composite were exhaustively examined. PCL, a well-known degradable biomaterial, has been extensively applied in biomedical applications 31. PCL can be fabricated into pretty smooth and uniform fibers, with a diameter of 5.4 ± 1.0 μm, *via* electrospinning as confirmed in **Figure S3A and S3B**. Interestingly, under the same electrospinning conditions, a thinner diameter of 2.4 ± 0.8 μm (**Figure 1A and S3C**) was detected on the Rop-loaded PCL fibers, which may be due to the high content of Rop in the fiber. Of note, compared to PCL fiber, the PCL-Rop fiber exhibited a cotton-like softness, exactly addressing the pitfall of PCL fiber with strong toughness which is insufficient as an injectable drug carrier. In addition, the extended PCL-Rop fiber with an even thinner diameter of 0.3 ± 0.1 μm upon mixing with F127 hydrogel and lyophilization was observed by SEM imaging (**Figure 1A and S3D**), which was a benefit for performing the injection. To get a clear vision of the two components of PCL-Rop fiber and F127 hydrogel in the injectable gel composite, FITC labeled Fiber-Rop (green channel) and Rhodamine B (RhB) labeled Clo-Gel (red channel) were captured by fluorescence microscope (**Figure 1B**), which not only indicated the good dispersity of the fiber in the gel phase but also implied the drug encapsulate capability of each phase as without obvious dye molecule diffusion to another phase. Taking the advantage of the temperature-sensitive F127 phase, the fluidic sol solution endowed a good dispersity of the immersed fiber and drug at 4 °C, subsequently, the relative solid gel solution prevented the fast diffusion and remained the designed shape of the injective composite at 37 °C as shown in **Figure 1C**. Actually, both F127 hydrogel and Fiber-Rop/Gel-Clo composite showed rapid sol-gel transition within a few seconds from 4 °C to body temperature (37 °C). As shown in **Figure 1D**, before gelation, the values of G′ and G″ of Gel-Clo was relatively low with G″ higher than G′, indicating a sol state of the system. Whereas the value of G′ was higher than G″ of the Fiber-Rop/Gel-Clo composite, implying the addition of fiber mainly affected the moduli in the sol state. With the temperature gradually climbing up, the gelation of Gel-Clo and Fiber-Rop/Gel-Clo composite appeared at 15.8 °C and 15.9 °C, respectively, indicating that the addition of Fiber-Rop did not significantly alter the temperature of hydrogel formation. Notably, the maximum values of G′ and G″ of Fiber-Rop/Gel-Clo composite upon gelation were higher than those of Gel-Clo, which indicated that Fiber-Rop/Gel-Clo composite possessed a superior stiffness (**Figure 1D**).

Benefited from the gelation of F127, the Fiber-Rop/Gel-Clo composite can not only maintain the shape upon post-injection into the nerve tissue to prevent the fast diffusion of the cargos, but also render the composite sustainable drug release behavior. In particular, *in vitro* drug release experiments were performed to investigate the controlled dual-drug release capacity of the Fiber/Gel composite. As shown in** Figure 1E and 1F**, Rop released slowly from the Fiber-Rop/Gel-Clo composite, presenting the cumulative release of 2.1 ± 0.4%, 7.8 ± 0.3%, 23.4 ± 0.8% and 86.1 ± 0.5% in 6 h, 10 h, 24 h and 14 days, respectively. In contrast, the release pattern of Clo followed the initial burst release and zero-order release rate, displaying the cumulative release of 57.3 ± 3.6 %, 90.8 ± 5.0%, 98.0 ± 2.0%, and 100% after 6 h, 10 h, 24 h and 14 days, respectively. In principle, the diffusion of F127 hydrogel contributes to the burst release of Clo. To confirm the diffusion of F127 hydrogel, the morphology of RhB stained hydrogel was monitored over time (**Figure S4C**), which was visually and experimentally evidenced by the relatively fast diffusion in 24 h. Consequently, the rapid diffusion of F127 hydrogel was exactly in accord with our expectation that fast-released Clo from the gel could shrink the arterial diameter to prevent the absorption of Rop into the bloodstream and enhance the anesthetic effect. In addition, to simulate the Rop and Clo release from the composite under the relative biological environment with enzyme, lipase was introduced into the buffer among degradation observation, which promoted the degradation of the PCL fiber accompanied by faster drug release manners (**Figure 1E and 1F**). These results validated that Clo and Rop achieved sequential and sustained release profiles predominately resulting from the biodegradability of the composites *in vitro*, indicating the great potential in synergistically anesthetic effect *in vivo*.

Collectively, it was well demonstrated that we had successfully fabricated an injectable, temperature-sensitive, high drug-loaded Fiber-Rop/Gel-Clo composite with sequential drug release behavior, providing a feasible strategy for promoting anesthetic effect *in vivo*.

### In vivo effect of sciatic nerve blockade

**Figure 2:**
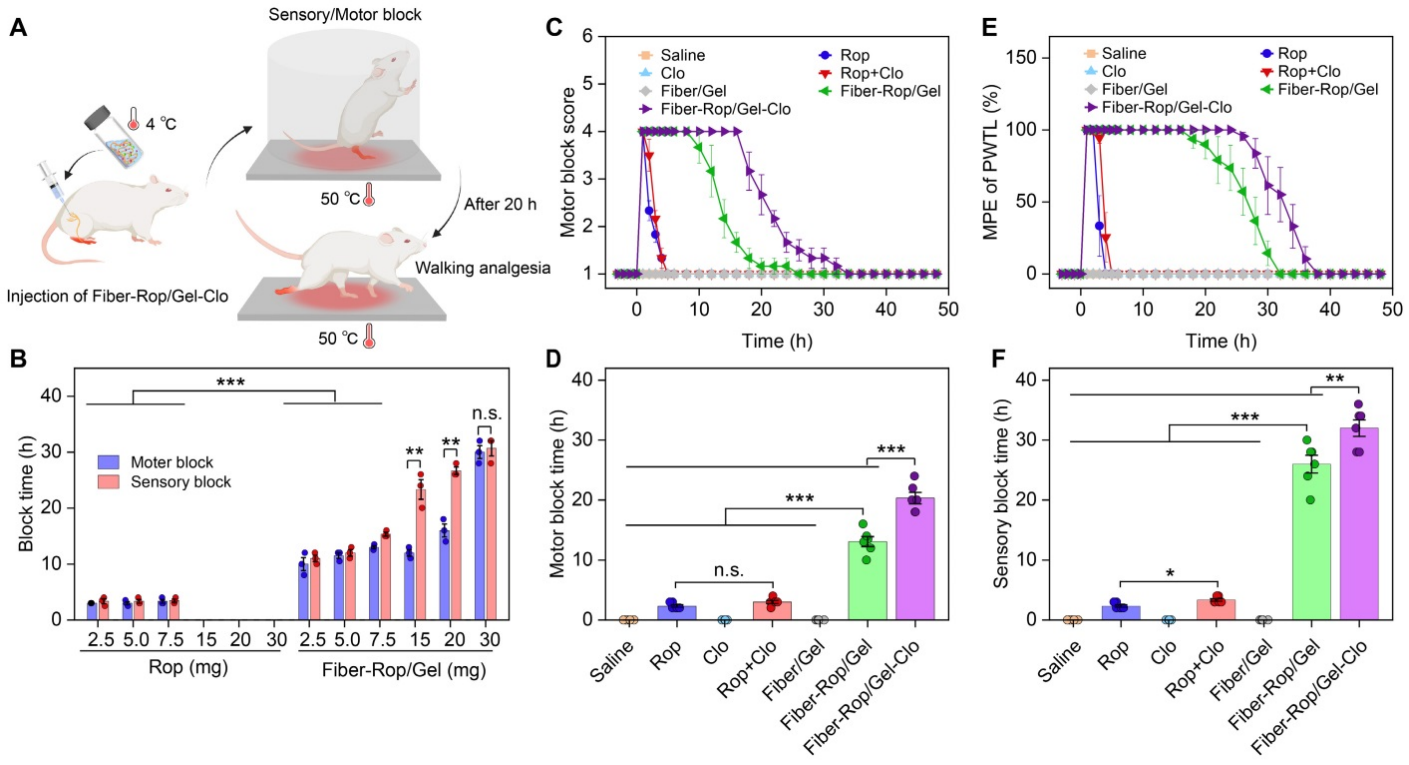
** Evaluation of sciatic nerve blockade effect in rats.** (**A**) Schematic diagram of rat sciatic nerve block. Created with BioRender.com. (**B**) Comparison of the sciatic nerve blockade effect in rats after different doses of free Rop solution or Fiber-Rop/Gel composite injection. Data are shown as mean ± standard error, (n = 3/group, n.s, not significant; **P < 0.01, ***P < 0.001). (**C**) Motor block was evaluated with a four-point rating scale. (**D**) Duration of motor block was the time from score 4 to score 2. (**E**) Sensory block was determined by paw withdrawal thermal latency (PWTL), and percentage of maximum possible effect (%MPE) was calculated as the effect of sensory blockade. (**F**) Duration of sensory block was the time from %MPE reaching 50% to less than 50%. Data are shown as mean ± standard error, (n = 6/group; n.s, not significant; **P < 0.01, ***P < 0.001).

In order to evaluate the anesthetic effect, various formulas were injected into the sciatic nerve of rats as illustrated in **Figure 2A**. Primarily, to determine the optimal dose of Rop, the anesthetic effect was accessed in the free Rop group and Fiber-Rop/Gel group, respectively, as a function of Rop dosage spanning from 2.5 mg to 30 mg. The nerve block time presented as approximately 2-3 h with no obvious difference upon administration of 2.5 mg, 5.0 mg, and 7.5 mg free Rop (**Figure 2B**). Meanwhile, the local anesthetic intoxication was under careful observation after perineural injection into rats placed on a hot plate. Notably, as soon as the Rop (0.5%, wt%) dosage increased up to 15 mg, the rats represented the symptoms of limb paralysis, convulsions, respiratory depression, and eventually died (**Video S4**). In contrast, the Fiber-Rop/Gel composite loaded with the same dose of Rop, showed merely unilateral sciatic nerve block without other abnormalities at the onset of the nerve blockage (**Video S5**). In addition, compared to the free Rop treated rats, the anesthetic effect of Fiber-Rop/Gel composite with the same Rop dose, from 2.5 mg to 30 mg, was pronouncedly promoted confirmed by much longer nerve block time as plotted in **Figure 2B**. Also, there were no obvious differences between motor block time and sensory block time upon administration of the Fiber-Rop/Gel composite at Rop doses of 2.5 mg, 5.0 mg, and 7.5 mg. To further prolong the nerve blockade duration by the composites, we gradually increased the percentage of Fiber-Rop in the hydrogel composite with maintaining the injectability. As shown in **Figure 2B**, the sensory block time was significantly extended step by step with increasing the Rop dose in the composite. For instance, the motor block and sensory block time of Fiber-Rop/Gel composite loading with 20 mg Rop specifically achieved up to 16.0 ± 0.8 h and 25.8 ± 1.0 h, respectively, and more importantly with rats exhibiting good conscious state and unobserved anesthetic intoxication (**Video S6**, 48 h post-injection). Remarkably, the sensorimotor segregation effect was observed immediately until the motor block expired upon administration of the Fiber-Rop/Gel composite loading with 15 mg, and 20 mg Rop. It should be pointed out that sensorimotor segregation is extremely important in clinical surgery 32, 33. According to our observation, the blocked foot of the rats could walk normally without any response to the hot plate, which directly and visually confirmed the striking sensorimotor segregation effect. However, the sensorimotor segregation effect was not strengthened until increasing the Rop dose up to 30 mg in the composite. Specifically, the motor and sensory blockade were 28.0 ± 2.2 h and 29.5 ± 1.5 h, respectively, upon administration of the 30 mg Rop-loaded Fiber-Rop/Gel composite, with ignorable separation of motor and sensory blockage. In addition, the high content of Fiber-Rop became impeding the smooth injection of the composite into rats. Taken together, 20 mg Rop in the Fiber-Gel composite was determined as the optimal dosage with satisfactory block time and optimum sensorimotor segregation effect, which was employed in the further experiments.

Next, we embarked on investigating the detailed course of the sensorimotor response and segregation upon administration of the six formulations presented in** Figure 2C-2F**. Based on the exploration of Rop dosage-dependent anesthetic effect, saline, 5 mg Rop in the free drug group, 2.5 μg Clo as adjuvant, 5 mg Rop + 2.5 μg Clo (Rop:Clo = 2000:1, wt%), blank Fiber/Gel composite, 20 mg Rop-loaded Fiber-Rop/Gel composite, and 20 mg Rop and 10 μg Clo-loaded Fiber-Rop/Gel-Clo composite were primarily determined, considering of the safety and optimal motor and sensory blockade. As shown in **Figure 2C**, the groups of Rop, Rop + Clo, Fiber-Rop/Gel, and Fiber-Rop/Gel-Clo exhibited expected nerve blockade in the initial 10 min post injection of the drug. However, rats treated with saline, Clo alone, or blank Fiber/Gel composite showed no nerve block effect at all. The motor blockade in the Fiber-Rop/Gel (13.1 ± 0.8 h) and Fiber-Rop/Gel-Clo (20.3 ± 0.9 h) groups lasted significantly longer than that in the free Rop (2.3 ± 0.2 h) and Rop + Clo (3.0 ± 0.3 h) groups (*P* < 0.001), clearly indicating that the controlled release of Rop from the Fiber/Gel composite dramatically prolonged the nerve block duration (**Figure 2C and 2D**). Notably, with the assistance of Clo, Fiber-Rop/Gel-Clo markedly prolonged the nerve block effect than Fiber-Rop/Gel (*P* < 0.01).

In addition, the entire process of sensory block was carefully monitored as well, in which the paw withdrawal thermal latency (PWTL) was observed over time and the percentage of maximum possible effect (MPE) was semi-quantitatively recorded on behalf of the effect of sensory blockade (**Figure 2E and 2F**). Likewise, despite saline, Clo alone, or blank Fiber/Gel composite groups with the undetectable sensory blockade, the sensory blockade occurred in Rop, Rop + Clo, Fiber-Rop/Gel, and Fiber-Rop/Gel-Clo groups with effective duration time of 2.3 h, 3.3 h, 26.0 h, and 32.0 h, respectively. Strikingly, the sensory block time sustained much longer (26.0 h vs 13.1 h; 32.0 h vs 20.3 h) than the motor block time in Fiber-Rop/Gel and Fiber-Rop/Gel-Clo groups, respectively, demonstrating the evident sensorimotor segregation effect, which was extremely promising in translating the injectable drug-loaded composite from bench to clinic. Apparently, Clo contributed to the promotion of sensory blockade consistent with the effect in the motor blockade, resulting in the longer nerve block time post-injection of Fiber-Rop/Gel-Clo into the sciatic nerve site compared to Fiber-Rop/Gel.

### The mechanism of Clo for prolonged sciatic nerve blockade

**Figure 3:**
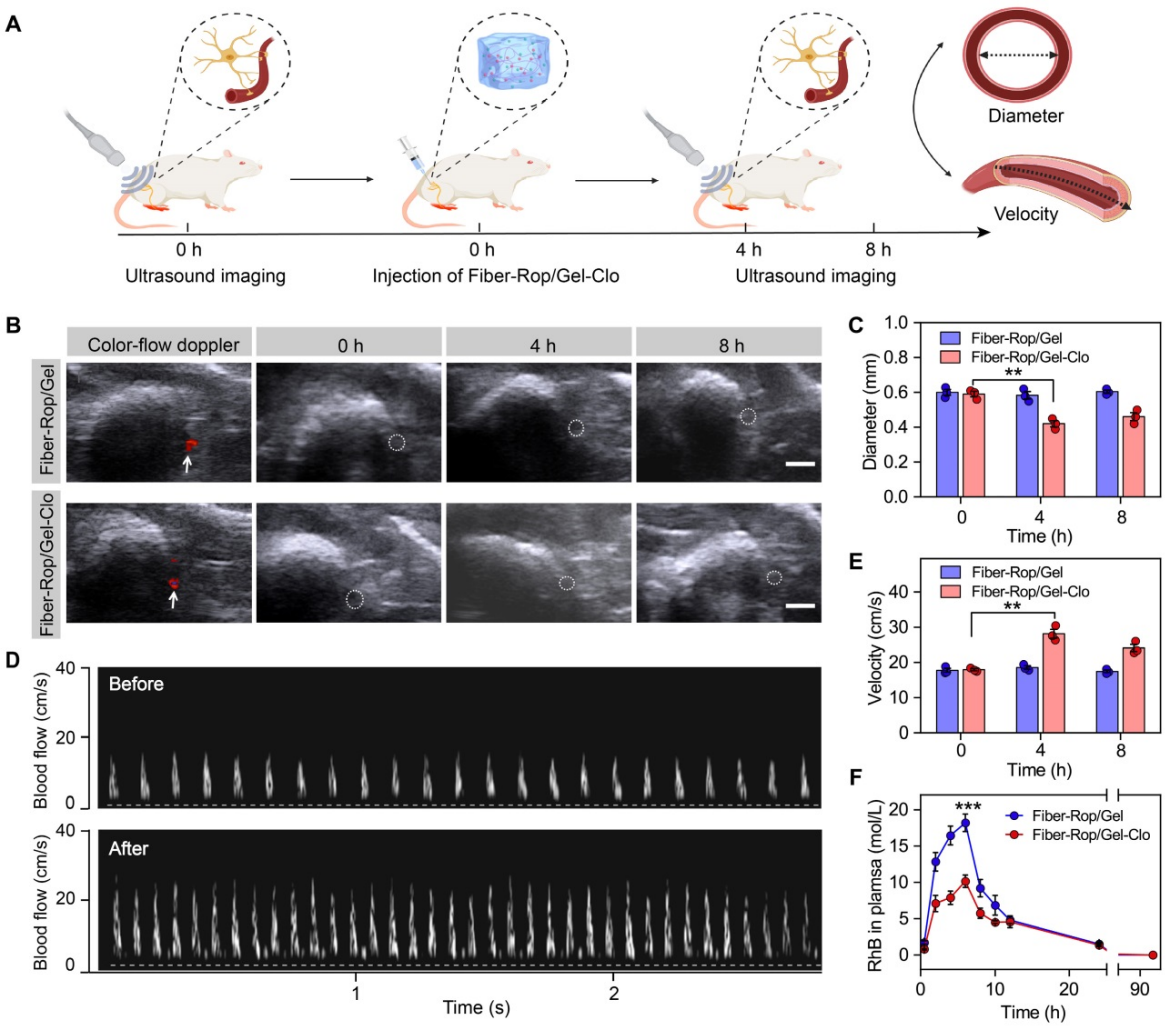
** Clo reduces the concentration of Rop absorbed by local vasoconstriction.** (**A**) Schematic diagram of the experimental program to evaluate the role of Clo by ultrasound imaging. Created with BioRender.com. (**B**) Representative images of arterial diameter around the sciatic nerve at 0 h, 4 h, and 8 h after injection of Fiber-Rop/Gel composites with or without Clo. White arrows show the location of blood vessel and white circles show the outline of blood vessel. Scale bar, 1.0 mm. (**C**) Qualitative analyses of arterial diameter around the sciatic nerve at 0 h, 4 h, and 8 h after injection of Fiber-Rop/Gel composites with or without Clo. (**D**) Representative images of blood flow velocity at 0 h and 4 h after injection of Fiber-Rop/Gel-Clo composites. (**E**) Qualitative analyses of blood flow velocity around the sciatic nerve at 0 h, 4 h, and 8 h after injection of Fiber-Rop/Gel composites with or without Clo. (**F**) The dynamic pharmacokinetics of RhB released from Fiber-Rop-RhB/Gel composite and Fiber-Rop-RhB/Gel-Clo composite in rats. Data are shown as mean ± standard error, (n = 3/group; **P < 0.01, ***P < 0.001).

The aforementioned results confirmed that the addition of Clo gave rise to prolonging the nerve block effect. To unveil the mechanism of Clo-assisted anesthetic effect, ultrasonography was used to detect the arterial diameter and blood flow velocity interfered by Clo addition around the sciatic nerve (**Figure 3A and Figure S5**). As depicted in **Figure 3B and 3C**, the arterial diameter displayed no obvious alteration post-injection of Fiber-Rop/Gel in the absence of Clo, with an average diameter of approximately 0.6 mm measured at the predetermined time points as 0 h, 4 h, and 8 h. In contrast, the addition of Clo with a trace amount (10 μg) significantly elicited decreased diameter of the artery at 4 h and 8 h, approaching 71.2% (0.42 mm/0.59 mm) and 77.9% (0.46 mm/0.59 mm) of the original arterial diameter (*P* < 0.01). Apparently, the addition of Clo predominately contributed to the constriction of the blood vessel. Afterwards, blood flow velocity was locally monitored over time, which was proved to be inversely proportional to the local vascular diameter according to Bernoulli's principle 34. It was obvious that the blood flow velocity significantly increased by 157.1% (28.17/17.93 cm/s) at 4 h post-injection of Fiber-Rop/Gel-Clo and slightly attenuated at 8 h (**Figure 3D and 3E**), compared to the velocity before administration. However, no obvious alteration of the flow velocity was detected in the examined blood vessels before and after injection of Fiber-Rop/Gel, and even no visible difference until a longer observation time. As a consequence, the introduced Clo resulted in local vasoconstriction, which might hamper the absorption of Rop into the bloodstream, yielding a relatively lower concentration of Rop in the vessels and preserving higher content of Rop in the periphery of the sciatic nerve. To confirm our assumption, RhB was applied to mimic the pharmacokinetics of Rop in the bloodstream, which was assembled as the electrospun fiber component of the Fiber-Rop/Gel and Fiber-Rop/Gel-Clo composite. As depicted in **Figure 3F**, the concentration of RhB in the plasma of Fiber-Rop/Gel-Clo group was dramatically lower than that of the Fiber-Rop/Gel group as the time spanned from composite injection to 96 h. The AUC value was 188.61 mol/L·h and 129.17 mol/L·h in the Fiber-Rop/Gel and Fiber-Rop/Gel-Clo group, respectively. Therefore, the addition of Clo could significantly diminish the local anesthetic absorption into the bloodstream, ensuing promoted biosafety and local analgesia efficacy of the Fiber-Rop/Gel-Clo composite.

### The in vivo evaluation of biodegradation and biosafety of Fiber-Rop/Gel-Clo composite

**Figure 4:**
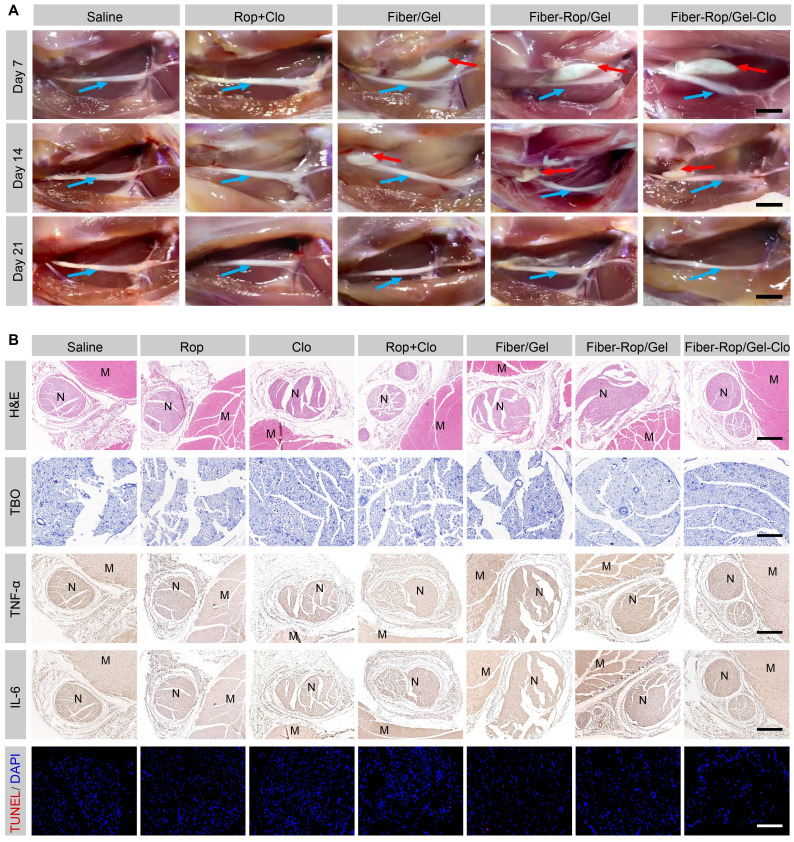
**Assessment of biodegradation and biosafety of Fiber-Rop/Gel-Clo composite in rats.** (**A**) *In vivo* degradation of Fiber/Gel composite. Local anatomical structures of sciatic nerves and surrounding muscles, and the potential composite remnants were captured on 7, 14, and 21 days after injection. Cyan arrows indicate sciatic nerves and red arrows indicate the composite remnants. Scale bar, 1 cm. (**B**) Hematoxylin and eosin (H&E) staining of sciatic nerves and surrounding muscles; scale bar, 500 μm. Toluidine blue O (TBO) staining of sciatic nerves; scale bar, 100 μm. Immunohistochemical analysis of inflammatory factors (TNF-α, IL-6) in the sciatic nerve and surrounding muscles; scale bar, 500 μm. TUNEL staining of sciatic nerves; scale bar, 100 μm. 'N' and 'M' represent the sciatic nerve and muscle, respectively.

It is essential to apply biodegradable materials for *in vivo* investigation, so that no severe side effects appear afterwards. To assess the biodegradation of the composite over time around the sciatic nerve, the injection site was dissected on day 7, 14, and 21 for gross examination. As seen from the local anatomical structure (**Figure 4A**), white composite remnants wrapped around the sciatic nerve were observed in all rats injected with Fiber/Gel, Fiber-Rop/Gel, and Fiber-Rop/Gel-Clo after 7 days. With gradual degradation of the composite, the residue became much smaller on day 14, and got completely degraded after 21 days. Of note, the degradation rate of Fiber-Rop/Gel-Clo composite was pretty slow in pure PBS *in vitro*, which was accelerated upon immersing the composite in buffer with lipase (for degradation of the ester group of PCL) as shown in **Figure S4**. Compared to the degradation *in vitro*, the composite degraded much faster post-injection *in vivo*, presumably, the bioenvironment with various kinds of enzymes facilitated the catalyzation of the PCL polymer fiber degradation 35. Also, due to the small proportion of PCL (PCL:Rop = 1:3, w/w), which was also one of the causes of rapid degradation.

To evaluate the biosafety of free drugs and Fiber/Gel composite with different formulas, the rats were euthanized on the 7th postoperative day, and their sciatic nerves and surrounding muscles were collected for H&E staining, TBO staining, immunohistochemistry assay, and TUNEL assay. As shown in **Figure 4B**, no obvious injury or necrosis of sciatic nerves and muscles were observed between groups. TBO staining also showed regularly and compactly arranged neurons with perineural tissue, and no degeneration or alteration in myelin structure was found in the Fiber-Rop/Gel-Clo group. Furthermore, there were slight apoptosis and inflammatory infiltration among all of the groups, indicating there was no additional injury from the composite treatment (**Figure 4B**). It has been reported that subtle inflammation after injection of anesthetic-loaded biomaterials could decline and disappear over time 9, therefore, our designed Fiber-Rop/Gel-Clo composite was available for *in vivo* experiments.

**Figure 5:**
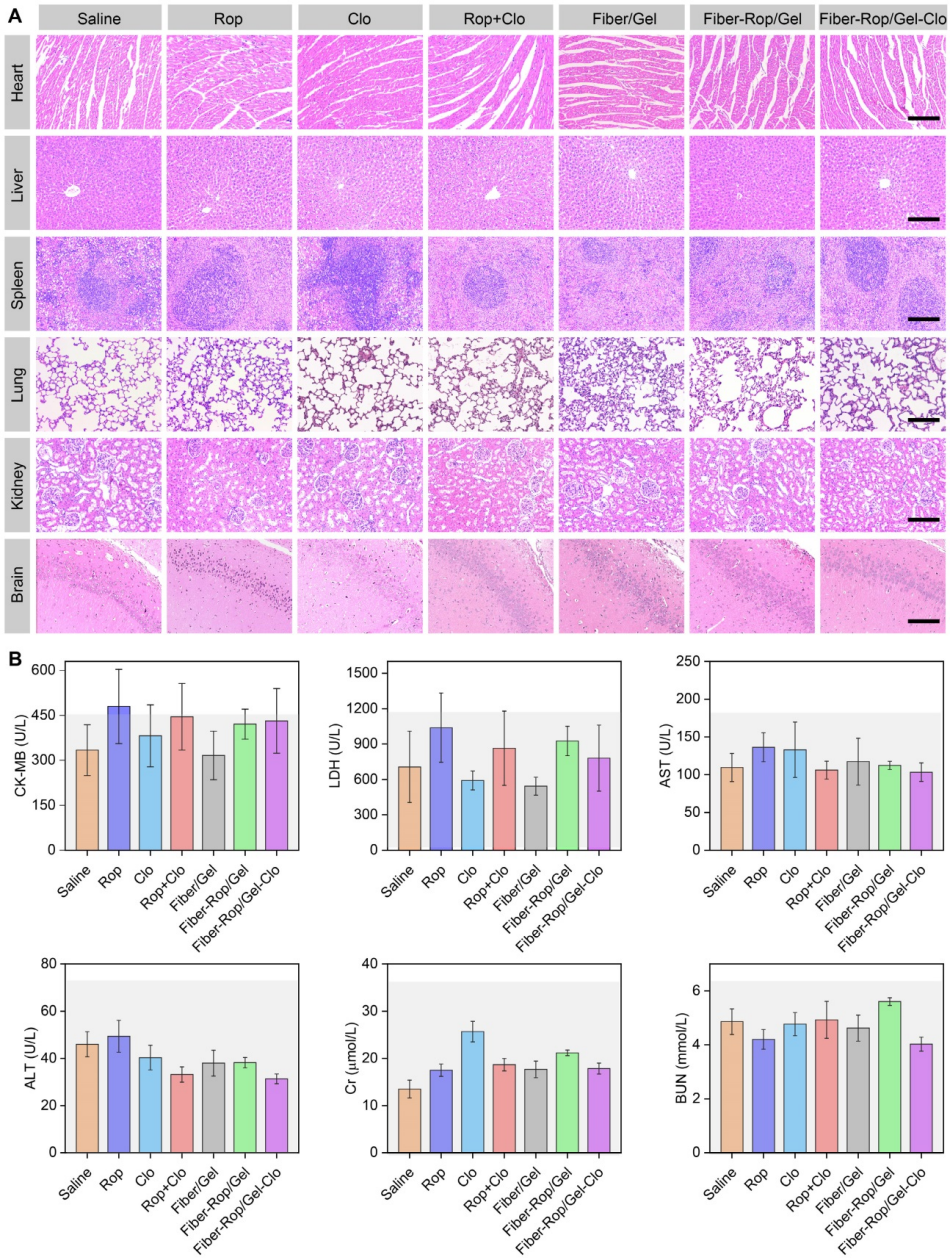
** Assessment of systemic toxicity of Fiber-Rop/Gel-Clo composite in rats.** (**A**) H&E staining of the heart, liver, spleen, lung, kidney, and brain (hippocampus CA1); scale bar, 200 μm. (**B**) The serum levels of CK-MB, LDH, AST, ALT, Cr, and BUN among the seven groups. The light gray backgrounds indicate the upper normal limit.

It is also of utmost importance to check the systemic toxicity of the injectable drug-loaded biomaterials, to ensure the biosafety of the drug delivery system. First of all, the mental state and body weight growth curves showed no difference between groups after injection, as well as no signs of local anesthetic intoxication (**Table S4, Figure S6**). Furthermore, six serological markers were detected to examine the heart (CK-MB, LDH), liver (AST, ALT), and kidney (Cr, BUN) functions, and no abnormal serum levels were observed in all seven groups 36 (**Figure 5B**). Additionally, no heart, liver, spleen, lung, kidney, and brain (hippocampus) injuries were found according to H&E staining (**Figure 5A and Figure S7**). Overall, it demonstrated that the Fiber-Rop/Gel-Clo composite possessed good biodegradability and biocompatibility, as well as negligible local and systemic toxicity *in vivo*.

## Discussion

Adequate analgesia is an integral part of patient recovery following surgery. Clinically, there are mainly four kinds of analgesic methods, including patient-controlled intravenous analgesia (PCIA), regional analgesia, topical infiltration analgesia, and oral analgesics 37. Among them, walking regional analgesia combines the rapid pain relief from the regional block whereas allows sufficient motor function for patients to ambulate 38. Thus, this method has been increasingly applied for postoperative analgesia, particularly for patients undergoing lower limb surgery (such as total knee arthroplasty), because it provides early ambulation, exactly superior analgesia, less narcotic analgesic drugs use, decreased postoperative nausea and vomiting, and decreased hospitalization cost. However, there is still no kind of local anesthetic, which could achieve long-lasting and sensory-selective regional analgesia after a single dose. In addition, the continuous infusion of local anesthetics through an indwelling catheter throws the risks of mechanical nerve injury and infections. Over the past few years, research on long-acting local anesthetic development has been focused on three major methods: developing new long-acting local anesthetic, exploring novel assisted adjuvants, and investigating delivery systems with a controlled release manner 39-41. The most widely used drugs, lidocaine, ropivacaine, and bupivacaine, whose anesthetic effects all last less than 8 h 42. Exparel (bupivacaine-loaded liposome) is the only commercially available drug delivery system with nanomaterials. However, this agent could only be used for incision infiltration, with additional drawbacks such as expensive price and time-consuming preparation 43. Local anesthetics combined with adjuvants (such as adrenaline, dexamethasone, and dexmedetomidine) are relatively convenient, but the prolonged analgesic effect is insufficiently satisfactory 44-46. In this study, we combined three approaches to develop a promising long-acting local anesthetic. Firstly, Rop, the most clinically used and relatively long-acting drug, was preferentially selected as a local anesthetic. Secondly, Clo was introduced as an efficient adjuvant. And finally, our designed injectable Fiber/Gel platform was exploited as a sustained-release carrier.

By acting on the voltage-gated Na^+^ channel in the nerve cell membrane to inhibit the Na^+^ influx and suppress the action potential, local anesthetics reversibly block nerve conduction and thus result in regional anesthesia. In recent years, the nociceptive-selective block effect has always been a well-marked hotspot with huge challenges in local anesthetic research 47. Previous studies reported QX-314 (N-ethyl-lidocaine), a permanently charged and membrane-impermeant lidocaine derivative, could generate nociceptive-selective local analgesia. QX-314 could selectively enter into nociceptors when co-administered with capsaicin, a transient receptor potential vanilloid 1 (TRPV1) channel agonist. However, QX-314 might induce myotoxicity and neurotoxicity at a high dose 10, 11. Currently, low concentration of Rop presents a sensorimotor segregation effect, which has been well recognized and widely applied in clinical practice 48, 49. Due to its insufficient hydrophilicity, the arrival time of ropivacaine to the gross motor nerve was delayed, resulting in the weakened absolute efficacy for motor block 50. Moreover, it has been verified that Rop is more sensitive to Aδ and C nerve fibers (sensory nerve fibers) than other traditional local anesthetics (lidocaine, bupivacaine), thus it forms the unique character of the sensorimotor segregation effect 51. In previous studies, some Rop-loaded biomaterials did not achieve a separation effect 52, 53, which may be related to the drug release mode. In our study, the Fiber-Rop/Gel-Clo composite loading with Rop, especially with 15 mg and 20 mg Rop, visually showed rapid onset and distinguished motor and sensory block for more than 32.0 h with negligible systemic toxicity. Strikingly, with the sustained release of Rop from the composite, the sensorimotor separation effect of Rop appeared and was enhanced with the aid of Clo, as sensory block time of 32.0 h and motor block time of 20.3 h, which was the remarkable breakthrough of this study.

Previous studies have reported that Clo could enhance and prolong the local anesthetic effect 54, but the mechanism is not fully clear yet. On one hand, Clo could interfere with voltage-gated Na^+^ and K^+^ channels involved in action potential production in spinal dorsal horn neurons, thus shortening the onset time of local anesthetics 55. On the other hand, as an α2 adrenergic receptor agonist, Clo could reduce local anesthetics absorption and systemic toxicity by constricting blood vessels *via* activating α2 receptors on peripheral vascular smooth muscle 56. The results in the current study also confirmed that the addition of Clo could reduce the absorption of local anesthetic into the bloodstream by local vasoconstriction, and consequently improve the safety and drug efficacy of local anesthetics.

Electrospinning is a well-known versatile technique for fabricating fibers derived from polymers, with diameters on the nano- to micro-scale 57. The electrospun fiber membranes, with high specific surface area, adjustable porosity, and flexibility, have been widely developed for tissue engineering, biomedical drug delivery, biomedical scaffolds, and so forth 58. Drug delivery by electrospun fibers possesses a pyramid of merits, including superior drug loading content and drug loading efficiency, controlled drug release ability, facile and cost-effective process *etc*. 27. To date, due to the solid property of the conventional electrospun fiber, open surgery remains the predominant option for the direct and efficient administration of the target lesion. Researchers have ever paid efforts to mince the electrospun fibers into tiny fragments with cryogenic grinding or mechanical cutting method 59, 60, followed by combining electrospun fibers with injectable hydrogel, to achieve injectable, minimally invasive administration. However, it was doubted that the mincing process might elicit the alteration of the release manner of the cargos from fibers 61. Instead, in this study, to circumvent the drawbacks of the complicated preparation of fragmented fibers, we innovatively created injectable, cotton-like Rop-loaded PCL fibers by comprehensively testing a series of electrospinning parameters working on different polymer materials. In principle, PCL itself exhibits substantial hardness and flexibility. Whereas, our elaborately designed PCL-Rop fiber was as soft as cotton candy with a high drug payload, due to the small proportion of PCL (PCL:Rop = 1:3, w/w), which was the most highlighting property for further injection. To promote the smoothness of the injection, a specific percentage of the injectable F127 hydrogel was introduced to the system while maintaining the structure of fiber intact, yielding a developed Fiber/Gel composite loading with drugs. Unlike the previous bupivacaine-loaded electrospun PLGA nanomembrane inserted at the open incision 8, the Fiber-Rop/Gel-Clo composite in the current study could be smoothly injected around the rat sciatic nerve *via* a 1.2 mm-sized injection needle with marginal invasion. Therefore, our designed injectable fiber platform was convenient in the application of minimally invasive surgery, holding great potential in clinical application.

One objective for developing novel local anesthetic system is to reduce unexpected side effects. Classic local anesthetics administered by the traditional method might exhibit local and systemic toxicity. Systemic toxicity is mainly caused by a high dose of local anesthetic, rich in blood vessels around the nerve tissue, or unexpected injections into blood vessels 62. Depending on the concentration of anesthetic in serum, patients may present with symptoms such as bradycardia, arrhythmia, convulsion, coma, or even cardiac arrest 63. Meanwhile, local anesthetic exhibits time and dose-dependent toxicity to a variety of tissues, for instance, nerves may swell or even necrose 64. In this study, local anesthetic intoxication was observed after perineural injection with ≥ 15 mg free Rop, nevertheless, the Fiber-Rop/Gel composite loading with the same dose of Rop showed efficient sciatic nerve block without abnormalities. The rational explanation was that Rop with a single large dose diffuses widely and was easily absorbed by blood vessels. In contrast, the Rop-loaded composite was anchored at the sciatic nerve after injection and sol-gel transition, yielding the confinement of sustainably released Rop in the local nerve site. Additionally, the local vasoconstriction resulting from Clo also reduces the concentration of Rop absorbed by the bloodstream, which further indicated our designed composite was advantageous in reducing the toxicity of local anesthetics. Given that the toxicity of the material itself also needs attention, our selected PCL and F127 are both FDA-certified biomedical materials with good biosafety and biodegradability 65, 66.

Of note, some limitations are worth investigating and addressing in future studies. Firstly, although the nerve block induced by Fiber-Rop/Gel-Clo composite lasted for approximately 32 h, it would be more conducive to postoperative analgesia in the clinic if it could be further extended to 72 h. Secondly, the sensorimotor separation effect of the composite appeared at the later stage of drug administration but was absent at the early stage, presumably due to the relatively fast drug release in the initial period, thus the formulation and structure of the drug delivery platform need to be further improved and optimized to achieve complete sensorimotor separation in the entire drug duration. Thirdly, novel biomaterial without gel-like nature is a requisite for spinal or epidural analgesia.

## Conclusions

In the present study, we developed an injectable Fiber-Rop/Gel-Clo composite as a local anesthetic delivery system, consisting of Rop-loaded PCL electrospun fiber and Clo-loaded thermosensitive F127 hydrogel, which was facilely assembled for prolonging and sensory-selective regional analgesia by merely one dose *via* sequentially releasing Clo and Rop. Preferentially, Clo was rapidly released from the hydrogel for vasoconstriction which not only diminished the absorption of Rop into the bloodstream to minimize the systemic toxicity but also concentrated Rop in the injection site to enhance the regional analgesia. Subsequently, the sustainably released Rop from the electrospun fiber dramatically prolonged the sciatic nerve block of rats with the aid of Clo. It was worth noting that the sensory block lasted significantly longer than the motor block upon injection of 1 mL Fiber-Rop/Gel-Clo composite containing 20 mg Rop and 10 µg Clo, as the duration time was 32.0 ± 1.4 h and 20.3 ± 0.9 h, respectively. Furthermore, our designed injectable Fiber-Rop/Gel-Clo composite exhibited good biodegradability as well as satisfactory biosafety. Consequently, the fabricated Fiber-Rop/Gel-Clo composite, possessing smooth injectability, minimal invasion effect, prolonged regional analgesia, and remarkable sensorimotor segregation effect, may provide a promising prospect for long-term regional walking analgesia, especially in clinical surgery of the knee or hip joint replacement.

## Supplementary Material

Supplementary figures, tables, video legends.

Supplementary video 1.

Supplementary video 2.

Supplementary video 3.

Supplementary video 4.

Supplementary video 5.

Supplementary video 6.

## Abbreviations

ALT: alanine transaminase
AST: aspartate aminotransferase
BUN: blood urea nitrogen
Cr: creatinine
Clo: clonidine
CK-MB: creatine kinase isoenzyme
DCM: dichloromethanemethanol
DLC: drug loading content
DLE: drug loading efficiency
DAB: diaminobenzidine
F127: Pluronic® F127
FITC: Fluorescein isothiocyanate
Fiber-Rop/Gel-Clo: ropivacaine-loaded poly (ε-caprolactone) electrospun fiber and clonidine-loaded F127 hydrogel
G′: storage modulus
G″: loss modulus
H_2_O_2_: hydrogen peroxide
LDH: lactate dehydrogenase
MPE: maximum possible effect
PBS: phosphate-buffered saline
PCL: Poly(ε-caprolactone)
PCIA: patient-controlled intravenous analgesia
PECG: poly (ethylene glycol)-block-poly (ε-caprolactone)-graft-poly (2-(guanidyl) ethyl methacrylate)
PLGA: poly (lactic-*co*-glycolic acid)
PLGA-PEG-PLGA: poly (DL-lactide-*co*-glycolide-*b*-ethylene glycol-*b*-DL-lactide-*co*-glycolide
PLA: polylactic acid
PNB: peripheral nerve block
PWTL: paw withdrawal thermal latency
QX-314: *N*-ethyl-lidocaine
Rop: ropivacaine
Rop-HCl: Ropivacaine hydrochloride
RhB: Rhodamine B
SE: standard error
TBO: Toluidine blue O dye
TUNEL: TdT mediated dUTP Nick End Labeling
TRPV1: transient receptor potential vanilloid 1

## References

[1] Kehlet H (2018). Postoperative pain, analgesia, and recovery-bedfellows that cannot be ignored. Pain.

[2] Rawal N (2016). Current issues in postoperative pain management. Eur J Anaesthesiol.

[3] Memtsoudis SG, Cozowicz C, Bekeris J, Bekere D, Liu J, Soffin EM (2021). Peripheral nerve block anesthesia/analgesia for patients undergoing primary hip and knee arthroplasty: Recommendations from the international consensus on anesthesia-related outcomes after surgery (icaros) group based on a systematic review and meta-analysis of current literature. Reg Anesth Pain Med.

[4] Petrini FM, Bumbasirevic M, Valle G, Ilic V, Mijović P, Čvančara P (2019). Sensory feedback restoration in leg amputees improves walking speed, metabolic cost and phantom pain. Nat Med.

[5] Leone S, Di Cianni S, Casati A, Fanelli G (2008). Pharmacology, toxicology, and clinical use of new long acting local anesthetics, ropivacaine and levobupivacaine. Acta Biomed.

[6] Joshi G, Gandhi K, Shah N, Gadsden J, Corman SL (2016). Peripheral nerve blocks in the management of postoperative pain: Challenges and opportunities. J Clin Anesth.

[7] Lirk P, Hollmann MW, Strichartz G (2018). The science of local anesthesia: Basic research, clinical application, and future directions. Anesth Analg.

[8] He Y, Qin L, Fang Y, Dan Z, Shen Y, Tan G (2020). Electrospun plga nanomembrane: A novel formulation of extended-release bupivacaine delivery reducing postoperative pain. Mater Des.

[9] Zhang W, Xu W, Ning C, Li M, Zhao G, Jiang W (2018). Long-acting hydrogel/microsphere composite sequentially releases dexmedetomidine and bupivacaine for prolonged synergistic analgesia. Biomaterials.

[10] Binshtok AM, Gerner P, Oh SB, Puopolo M, Suzuki S, Roberson DP (2009). Coapplication of lidocaine and the permanently charged sodium channel blocker QX-314 produces a long-lasting nociceptive blockade in rodents. Anesthesiology.

[11] Cheung HM, Lee SM, MacLeod BA, Ries CR, Schwarz SK (2011). A comparison of the systemic toxicity of lidocaine versus its quaternary derivative QX-314 in mice. Can J Anaesth.

[12] Banerjee M, Baranwal A, Saha S, Saha A, Priestley T (2015). En3427: A novel cationic aminoindane with long-acting local anesthetic properties. Anesth Analg.

[13] Vyas KS, Rajendran S, Morrison SD, Shakir A, Mardini S, Lemaine V (2016). Systematic review of liposomal bupivacaine (exparel) for postoperative analgesia. Plast Reconstr Surg.

[14] Ilfeld BM, Eisenach JC, Gabriel RA (2021). Clinical effectiveness of liposomal bupivacaine administered by infiltration or peripheral nerve block to treat postoperative pain. Anesthesiology.

[15] Markham A, Faulds D (1996). Ropivacaine. A review of its pharmacology and therapeutic use in regional anaesthesia. Drugs.

[16] Krishna Prasad GV, Khanna S, Jaishree SV (2020). Review of adjuvants to local anesthetics in peripheral nerve blocks: Current and future trends. Saudi J Anaesth.

[17] Mostafa MF, Hamed E, Amin AH, Herdan R (2021). Dexmedetomidine versus clonidine adjuvants to levobupivacaine for ultrasound-guided transversus abdominis plane block in paediatric laparoscopic orchiopexy: Randomized, double-blind study. Eur J Pain.

[18] Yang Y, Yu LY, Zhang WS (2018). Clonidine versus other adjuncts added to local anesthetics for pediatric neuraxial blocks: A systematic review and meta-analysis. J Pain Res.

[19] Brigham NC, Nofsinger R, Luo X, Dreger NZ, Abel AK, Gustafson TP (2021). Controlled release of etoricoxib from poly(ester urea) films for post-operative pain management. J Control Release.

[20] Zheng C, Li M, Ding J (2021). Challenges and opportunities of nanomedicines in clinical translation. BIO Integration.

[21] Li S, Jiang W, Zheng C, Shao D, Liu Y, Huang S (2020). Oral delivery of bacteria: Basic principles and biomedical applications. J Control Release.

[22] Zare Y, Shabani I (2016). Polymer/metal nanocomposites for biomedical applications. Mater Sci Eng C Mater Biol Appl.

[23] Correa S, Grosskopf AK, Lopez Hernandez H, Chan D, Yu AC, Stapleton LM (2021). Translational applications of hydrogels. Chem Rev.

[24] Zhou C, Huang J, Yang Q, Li T, Liu J, Qian Z (2018). Gold nanorods-based thermosensitive hydrogel produces selective long-lasting regional anesthesia triggered by photothermal activation of transient receptor potential vanilloid type-1 channels. Colloids Surf B Biointerfaces.

[25] Zhang W, Ning C, Xu W, Hu H, Li M, Zhao G (2018). Precision-guided long-acting analgesia by gel-immobilized bupivacaine-loaded microsphere. Theranostics.

[26] Foley PL, Ulery BD, Kan HM, Burks MV, Cui Z, Wu Q (2013). A chitosan thermogel for delivery of ropivacaine in regional musculoskeletal anesthesia. Biomaterials.

[27] Chen S, Li R, Li X, Xie J (2018). Electrospinning: An enabling nanotechnology platform for drug delivery and regenerative medicine. Adv Drug Deliv Rev.

[28] Feng X, Li J, Zhang X, Liu T, Ding J, Chen X (2019). Electrospun polymer micro/nanofibers as pharmaceutical repositories for healthcare. J Control Release.

[29] Bajwa SJ, Bajwa SK, Kaur J (2010). Comparison of epidural ropivacaine and ropivacaine clonidine combination for elective cesarean sections. Saudi J Anaesth.

[30] Shikanov A, Domb AJ, Weiniger CF (2007). Long acting local anesthetic-polymer formulation to prolong the effect of analgesia. J Control Release.

[31] Malikmammadov E, Tanir TE, Kiziltay A, Hasirci V, Hasirci N (2018). PCL and PCL-based materials in biomedical applications. J Biomater Sci Polym Ed.

[32] Jiménez-Almonte JH, Wyles CC, Wyles SP, Norambuena-Morales GA, Báez PJ, Murad MH (2016). Is local infiltration analgesia superior to peripheral nerve blockade for pain management after THA: A network meta-analysis. Clin Orthop Relat Res.

[33] You D, Qin L, Li K, Li D, Zhao G, Li L (2021). A meta-analysis on advantages of peripheral nerve block post-total knee arthroplasty. Korean J Pain.

[34] Zhao X, Wang Y, Wait E, Mankowski W, Bjornsson CS, Cohen AR (2021). 3D image analysis of the complete ventricular-subventricular zone stem cell niche reveals significant vasculature changes and progenitor deficits in males versus females with aging. Stem Cell Reports.

[35] Khan I, Nagarjuna R, Dutta JR, Ganesan R (2019). Enzyme-embedded degradation of poly(ε-caprolactone) using lipase-derived from probiotic lactobacillus plantarum. ACS Omega.

[36] Feng LX, Zhao F, Liu Q, Peng JC, Duan XJ, Yan P (2020). Role of Nrf2 in lipopolysaccharide-induced acute kidney injury: Protection by human umbilical cord blood mononuclear cells. Oxid Med Cell Longev.

[37] Meng Y, Jiang H, Zhang C, Zhao J, Wang C, Gao R (2017). A comparison of the postoperative analgesic efficacy between epidural and intravenous analgesia in major spine surgery: A meta-analysis. J Pain Res.

[38] Mann C, Ouro-Bang'na F, Eledjam JJ (2005). Patient-controlled analgesia. Curr Drug Targets.

[39] Bhansali D, Teng SL, Lee CS, Schmidt BL, Bunnett NW, Leong KW (2021). Nanotechnology for pain management: Current and future therapeutic interventions. Nano Today.

[40] Ji T, Li Y, Deng X, Rwei AY, Offen A, Hall S (2021). Delivery of local anaesthetics by a self-assembled supramolecular system mimicking their interactions with a sodium channel. Nat Biomed Eng.

[41] Svirskis D, Procter G, Sharma M, Bhusal P, Dravid A, MacFater W (2020). A non-opioid analgesic implant for sustained post-operative intraperitoneal delivery of lidocaine, characterized using an ovine model. Biomaterials.

[42] Casati A, Putzu M (2005). Bupivacaine, levobupivacaine and ropivacaine: Are they clinically different?. Best Pract Res Clin Anaesthesiol.

[43] Burbridge M, Jaffe RA (2015). Exparel®: A new local anesthetic with special safety concerns. Anesth Analg.

[44] Schotanus MGM, Bemelmans YFL, van der Kuy PHM, Jansen J, Kort NP (2017). No advantage of adrenaline in the local infiltration analgesia mixture during total knee arthroplasty. Knee Surg Sports Traumatol Arthrosc.

[45] Sakamoto B, Harker G, Eppstein AC, Gwirtz K (2016). Efficacy of local anesthetic with dexamethasone on the quality of recovery following total extraperitoneal bilateral inguinal hernia repair: A randomized clinical trial. JAMA Surg.

[46] Ouchi K (2020). Dexmedetomidine 2 ppm is appropriate for the enhancement effect of local anesthetic action of lidocaine in inferior alveolar nerve block: A preliminary, randomized cross-over study. Clin J Pain.

[47] Jayakar S, Shim J, Jo S, Bean BP, Singeç I, Woolf CJ (2021). Developing nociceptor-selective treatments for acute and chronic pain. Sci Transl Med.

[48] Shah NA, Jain NP (2014). Is continuous adductor canal block better than continuous femoral nerve block after total knee arthroplasty? Effect on ambulation ability, early functional recovery and pain control: A randomized controlled trial. J Arthroplasty.

[49] Shah NA, Jain NP, Panchal KA (2015). Adductor canal blockade following total knee arthroplasty-continuous or single shot technique? Role in postoperative analgesia, ambulation ability and early functional recovery: A randomized controlled trial. J Arthroplasty.

[50] Casati A, Santorsola R, Cerchierini E, Moizo E (2001). Ropivacaine. Minerva Anestesiol.

[51] Hansen TG (2004). Ropivacaine: A pharmacological review. Expert Rev Neurother.

[52] Santamaria CM, Woodruff A, Yang R, Kohane DS (2017). Drug delivery systems for prolonged duration local anesthesia. Mater Today.

[53] He Y, Qin L, Huang Y, Ma C (2020). Advances of nano-structured extended-release local anesthetics. Nanoscale Res Lett.

[54] Koyyalamudi V, Sen S, Patil S, Creel JB, Cornett EM, Fox CJ (2017). Adjuvant agents in regional anesthesia in the ambulatory setting. Curr Pain Headache Rep.

[55] Nguyen V, Tiemann D, Park E, Salehi A (2017). Alpha-2 agonists. Anesthesiol Clin.

[56] Kumamoto E (2020). Inhibition of fast nerve conduction produced by analgesics and analgesic adjuvants-possible involvement in pain alleviation. Pharmaceuticals.

[57] Zhao J, Cui W (2020). Functional electrospun fibers for local therapy of cancer. Adv Fiber Mater.

[58] Xue J, Wu T, Dai Y, Xia Y (2019). Electrospinning and electrospun nanofibers: Methods, materials, and applications. Chem Rev.

[59] Li X, Cho B, Martin R, Seu M, Zhang C, Zhou Z (2019). Nanofiber-hydrogel composite-mediated angiogenesis for soft tissue reconstruction. Sci Transl Med.

[60] Li X, Zhang C, Haggerty AE, Yan J, Lan M, Seu M (2020). The effect of a nanofiber-hydrogel composite on neural tissue repair and regeneration in the contused spinal cord. Biomaterials.

[61] Lee S, Yun S, Park KI, Jang JH (2016). Sliding fibers: Slidable, injectable, and gel-like electrospun nanofibers as versatile cell carriers. ACS Nano.

[62] Neal JM, Barrington MJ, Fettiplace MR, Gitman M, Memtsoudis SG, Mörwald EE (2018). The third american society of regional anesthesia and pain medicine practice advisory on local anesthetic systemic toxicity: Executive summary 2017. Reg Anesth Pain Med.

[63] Groban L (2003). Central nervous system and cardiac effects from long-acting amide local anesthetic toxicity in the intact animal model. Reg Anesth Pain Med.

[64] Verlinde M, Hollmann MW, Stevens MF, Hermanns H, Werdehausen R, Lirk P (2016). Local anesthetic-induced neurotoxicity. Int J Mol Sci.

[65] Labet M, Thielemans W (2009). Synthesis of polycaprolactone: A review. Chem Soc Rev.

[66] Shriky B, Kelly A, Isreb M, Babenko M, Mahmoudi N, Rogers S (2020). Pluronic f127 thermosensitive injectable smart hydrogels for controlled drug delivery system development. J Colloid Interface Sci.

