# Supplementary material for: Injectable electrospun fiber-hydrogel composite sequentially releasing clonidine and ropivacaine for prolonged and walking regional analgesia
Source: Theranostics. 2022 Jun 21;12(11):4904–21. doi: 10.7150/thno.74845 (PMC9274753; doi:10.7150/thno.74845)
Supplement: Supplementary file 1 — Supplementary figures, tables, video legends. [file thnov12p4904s1.pdf]

Supplementary Material

Injectable electrospun fiber-hydrogel composite sequentially releasing clonidine and ropivacaine for prolonged and walking regional analgesia

Sufang Chen[1,3#], Weifeng Yao[1#], Haixia Wang[1,3#], Tienan Wang[1], Xue Xiao[1], Guoliang Sun[1], Jing Yang[1], Yu Guan[1], Zhen Zhang[1], Zhengyuan Xia[2], Mingqiang Li[1,3,4]*, Yu Tao[1,3]*, Ziqing Hei[1]*

1. Department of Anesthesiology and Center for Nanomedicine, The Third Affiliated Hospital, Sun Yat-sen University, Guangzhou 510630, China
2. Department of Medicine, The University of Hong Kong, Hong Kong 999077, China
3. Laboratory of Biomaterials and Translational Medicine, The Third Affiliated Hospital, Sun Yat-sen University, Guangzhou 510630, China
4. Guangdong Provincial Key Laboratory of Liver Disease Research, Guangzhou 510630, China

* Corresponding authors.

Email addresses: limq567@mail.sysu.edu.cn (M. Li), taoy28@mail.sysu.edu.cn (Y. Tao), heizq@mail.sysu.edu.cn (Z. Hei)

[#] Sufang Chen, Weifeng Yao, and Haixia Wang contributed equally to this work.

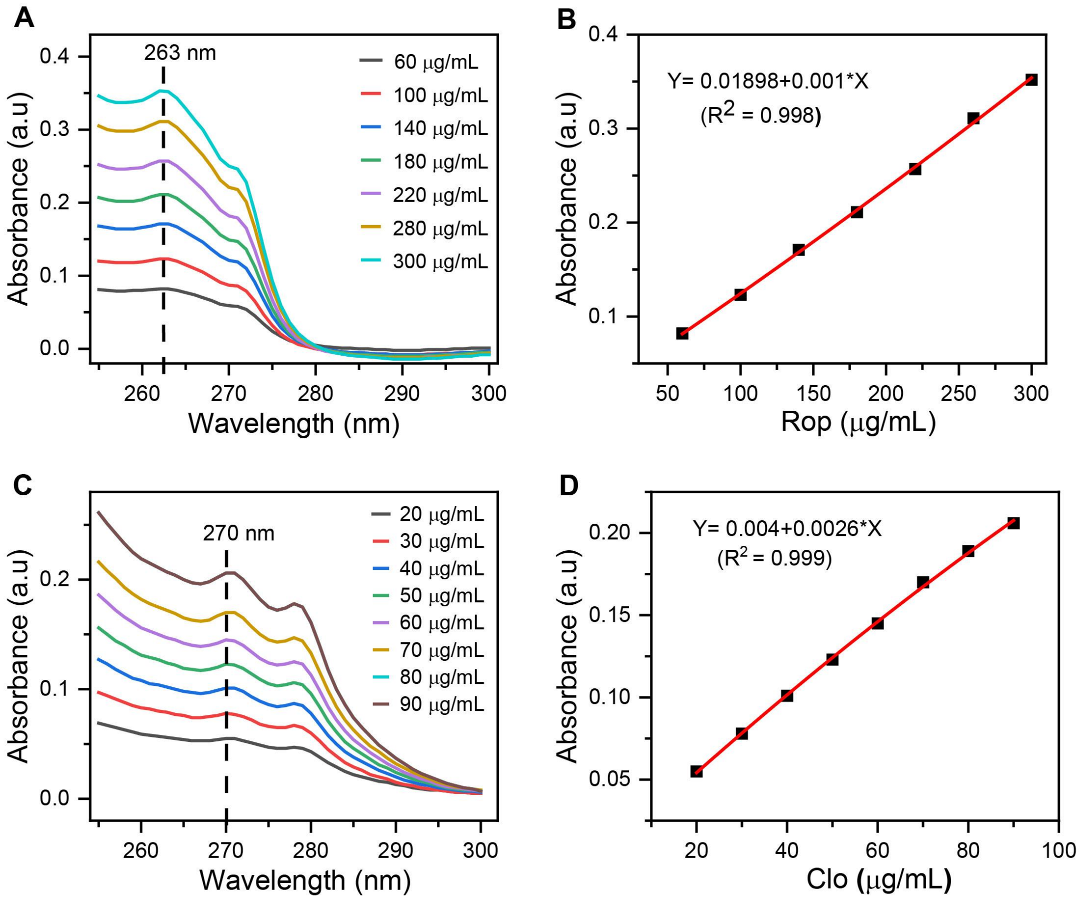

Figure S1. The absorbance of Rop and Clo detected by ultraviolet (UV). (**A**) Absorption curves of different concentrations of Rop in 250-300 nm wavelengths. (**B**) The linear calibration curves of Rop were calculated based on the maximum absorption value at 263 nm. (**C**) Absorption curves of different concentrations of Clo in 250-300 nm wavelengths. (**D**) The linear calibration curves of Clo were calculated based on the maximum absorption value at 270 nm.

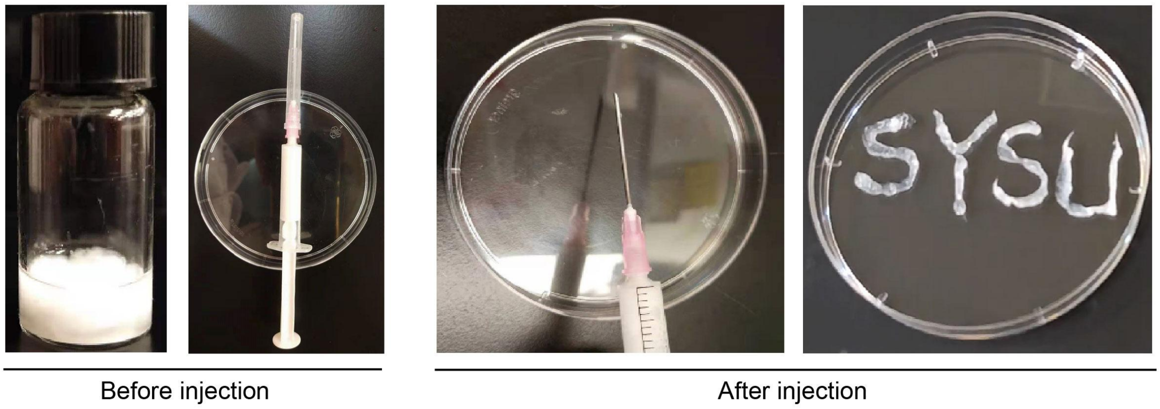

Figure S2. Photographs showing the Fiber-Rop/Gel-Clo composite before and after injection, which indicated the injective property of the composite.

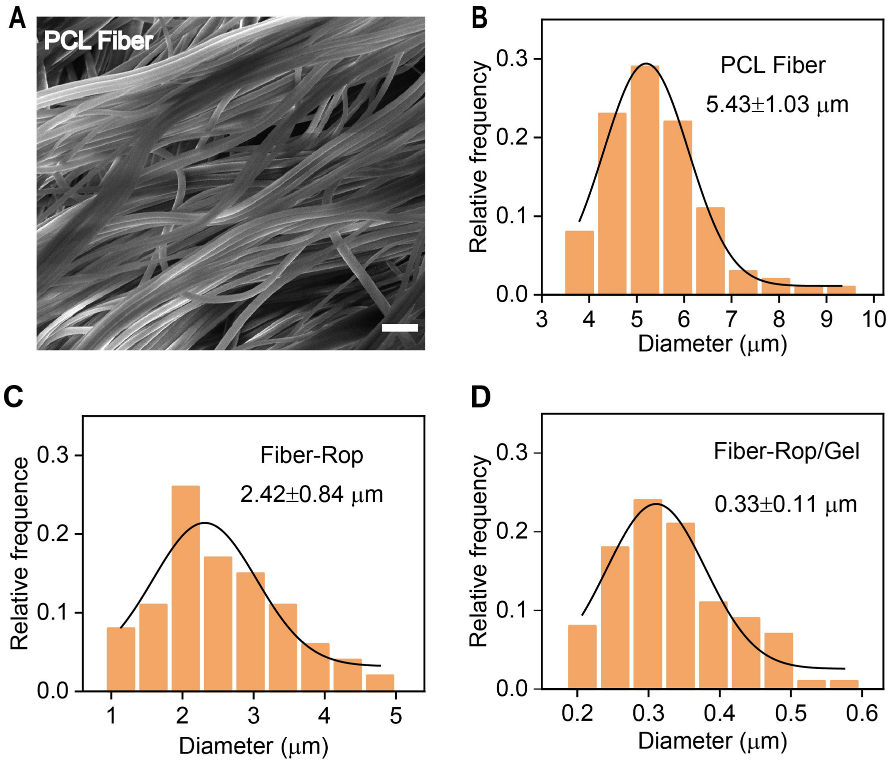

Figure S3. (**A**) Scanning electron microscopy (SEM) images of the PCL fiber; Scale bar, 10 μm. (**B-D**) The diameter distribution of PCL fiber, Rop-loaded fiber, and fiber in the Rop/Gel composite.

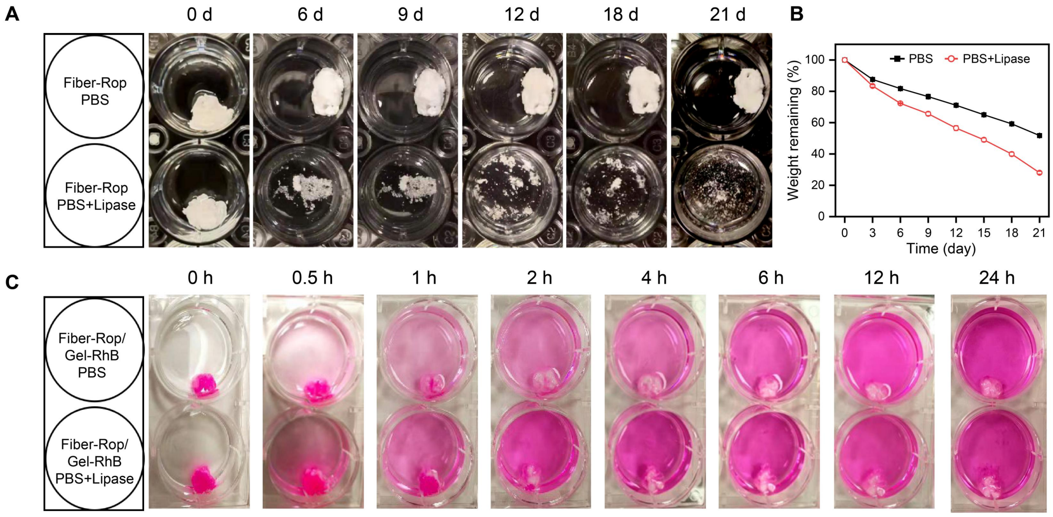

Figure S4. ***In vitro*** **degradation of Fiber-Rop/Gel-Clo composite.** (**A**) Photographs recording the morphology of Fiber-Rop in PBS solution with or without lipase (20 U/mL) at 0 day, 6 day, 9 day, 12 day, 18 day, and 21 day. (**B**) The initial weight (W_0) and remaining weight (W_1) at 0 day, 3 day, 6 day, 9 day, 12 day, 15 day, 18 day, and 21 day of the Fiber-Rop/Gel-Clo composites were recorded after lyophilizing for 24 h. Remaining weight (%) = $W_1/W_0 \times 100\%$. (**C**) Photographs recording the release of RhB from the Fiber-Rop/Gel-RhB composites in PBS solution with or without lipase (20 U/mL) at 0 h, 0.5 h, 1 h, 2 h, 4 h, 6 h, 12 h, and 24 h. Purple: RhB labeled gel; White: Fiber-Rop.

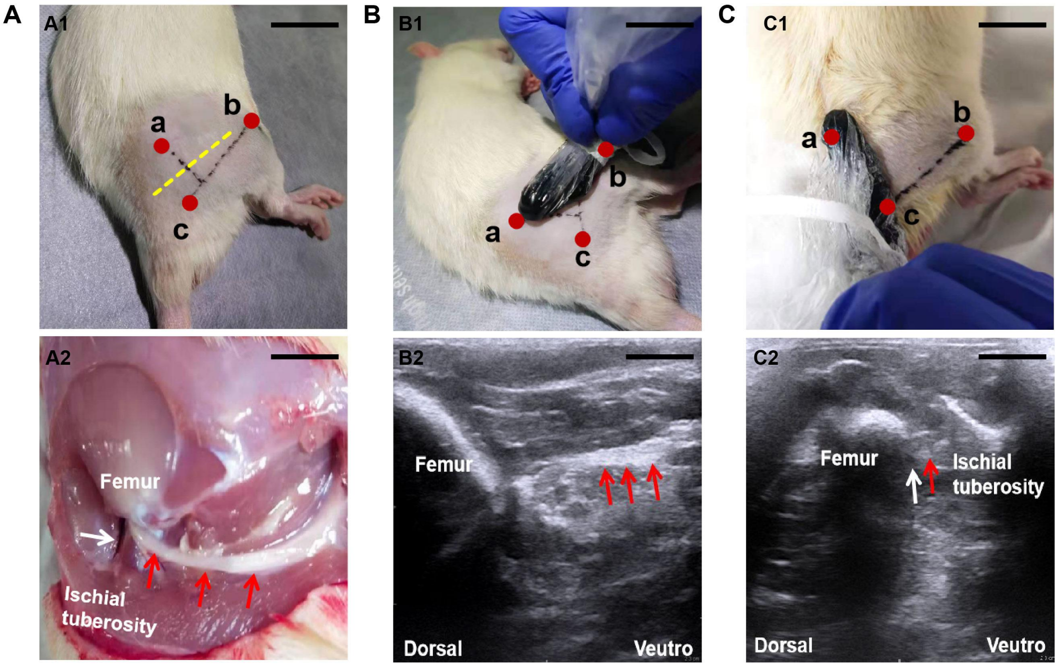

Figure S5. Anatomical structure and ultrasonography of the rat sciatic nerve. (**A**) Surface anatomical landmarks (A1) and gross pathology of the sciatic nerve (A2). The expected path of the sciatic nerve was marked with the yellow dotted line. The sciatic nerve runs between the femur (a) and ischial tuberosity (c), before branching off at the popliteal fossa (c). (**B**) Detection by the parallel placement of ultrasound probe (B1) to show the longitudinal image of the sciatic nerve (B2). (**C**) Detection by the transverse placement of ultrasound probe (C1) to show the cross-section image of the sciatic nerve and blood vessel (C2). The red arrows indicated the sciatic nerve, and the white arrows indicated the nearby blood vessel. **(A1), (B1), (C1)**: scale bar, 1 cm; **(A2), (B2), (C2)**: scale bar, 0.5 cm.

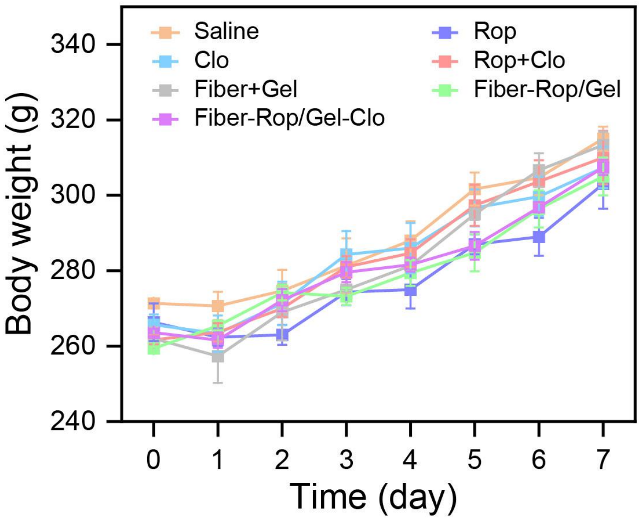

Figure S6. Body weight change of rats within one week after injection.

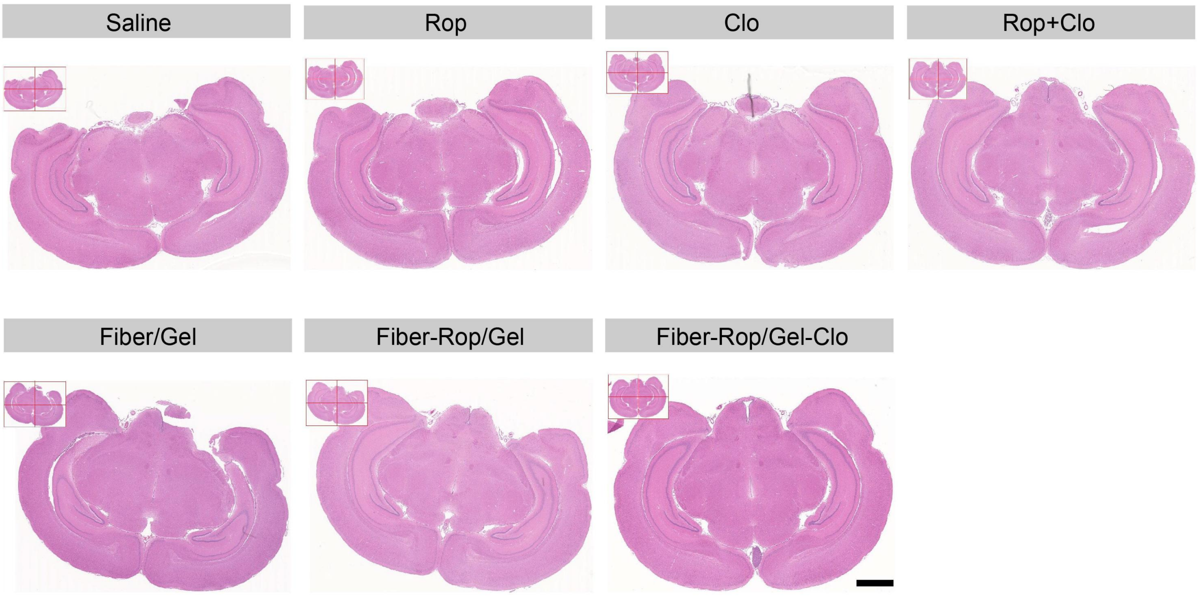

Figure S7. H&E staining of coronal sections of rat brain. Scale bar, 2 mm.

Table S1. Methods and parameters for producing stable and injectable electrospun nanofibers

Materials	Fiber (w/w)	Solvent	Parameters	Stable fiber	Injectable
PCL	10%PCL:Rop (3:1)	DCM	1.08 mL/h, 10 cm, 400 rpm, 9 kV	Yes	No
PCL	10%PCL:Rop (2:1)	DCM	1.08 mL/h, 10 cm, 400 rpm, 8 kV	Yes	No
PCL	10%PCL:Rop (1:1)	DCM	1.08 mL/h, 10 cm, 400 rpm, 8 kV	Yes	No
PCL	15%PCL:Rop (1:1)	DCM	1.08 mL/h, 10 cm, 400 rpm, 9.5kV	No	No
PCL	20%PCL:Rop (1:1)	DCM	1.08 mL/h, 10 cm, 400 rpm, 8.5kV	Yes	No
PCL	20%PCL:Rop (1:2)	DCM	1.08 mL/h, 10 cm, 400 rpm, 9 kV	Yes	No
**PCL**	**20%PCL:Rop (1:3)**	**DCM**	**1.08 mL/h, 10 cm, 400 rpm, 9 kV**	**Yes**	**Yes**
PCL	30%PCL:Rop (1:3)	DCM	1.08 mL/h, 10 cm, 400 rpm, 10 kV	No	No
PLGA	10%PLGA+Rop (3:1)	HFIP	1.08 mL/h, 7 cm, 400 rpm, 14 kV	No	No
PLGA	10%PLGA+Rop (2:1)	DCM	1.08 mL/h, 10 cm, 400 rpm, 12 kV	No	No
PLGA	10%PLGA+Rop (1:1)	DCM	1.08 mL/h, 10 cm, 400 rpm, 9 kV	No	No
PLGA	10%PLGA+Rop (1:2)	DCM	1.08 mL/h, 10 cm, 400 rpm, 8 kV	No	No
PLGA	15%PLGA+Rop (1:1)	DCM	1.08 mL/h, 11 cm, 400 rpm, 10 kV	No	No
PLGA	20%PLGA+Rop (1:1)	DCM	1.08 mL/h, 10 cm, 400 rpm, 9 kV	No	No
PLGA	25%PLGA+Rop (3:1)	HFIP	1.08 mL/h, 7 cm, 400 rpm, 15 kV	No	No
PLGA	30%PLGA+Rop (3:1)	HFIP	1.08 mL/h, 10 cm, 400 rpm, 8 kV	Yes	No
PLGA	30%PLGA+Rop (1:1)	HFIP	1.08 mL/h, 10 cm, 400 rpm, 15 kV	No	No

PLGA	30%PLGA+Rop (1:1)	HFIP+DCM (v/v 1:1)	1.08 mL/h, 10 cm, 400 rpm, 8 kV	No	No
PLGA	40%PLGA+Rop (1:1)	HFIP	1.08 mL/h, 10 cm, 400 rpm, 8 kV	No	No
PLA	2% PLA+ Rop (2:1)	HFIP	1.08 mL/h, 10 cm, 400 rpm, 8 kV	Yes	No
PLA	4% PLA+ Rop (1:1)	HFIP	1.08 mL/h, 10 cm, 400 rpm, 8 kV	Yes	No
PLA	6% PLA+ Rop (1:1)	HFIP	1.08 mL/h, 8 cm, 400 rpm, 10 kV	Yes	No
PLA	8%PLA+Rop (3:1)	HFIP	1.08 mL/h, 10 cm, 400 rpm, 10 kV	No	No
PLA	10%PLA+Rop (3:1)	HFIP	1.08 mL/h, 10 cm, 400 rpm, 12 kV	No	No

Rop: Ropivacaine; PCL: poly(ε-caprolactone); PLGA: poly(lactic-co-glycolic acid); PLA: polylactic acid; DCM: dichloromethane; HFIP: hexafluoroisopropanol.

'Paramaters: 1.08 mL/h, 10 cm, 400 rpm, 9 kV ' means that the solution was injected at a controlled rate of 1.08 mL/h, and a steady spindle-like jet of fibers was formed at 9 kV electrostatic field. The distance between the needle and the rotating collector (running at 400 rpm) was 10 cm.

Table S2. Drug loading content and drug loading efficiency of electrospun fibers

(n = 3)	Drug loading content (Mean ± standard error)	Drug loading efficiency (Mean ± standard error)
**Fiber-Rop (w/w = 1:3)**	72.4 ± 0.4%	96.5 ± 0.6%

Table S3. Methods for producing thermo-sensitive and injectable hydrogel

	**Hydrogel architecture (wt%)**	**Time to form hydrogel at room temperature (24 ± 0.5 °C)**	**Time to form hydrogel at 37 °C**
F127	20% F127	no	no
F127	25% F127	no	5 min
F127	30% F127	10 min	20 s
F127	35% F127	4 min	10 s
**F127**	**40% F127**	**2 min**	**immediate**

Table S4. Rats mental state and survival rate in one week after sciatic nerve block

Group	Mental state	Local anesthetic intoxication rate	7-day survival rate
**Saline**	Normal	0	100%
**Rop**	Normal	0	100%
**Clo**	Normal	0	100%
**Rop+Clo**	Normal	0	100%
**Fiber/Gel**	Normal	0	100%
**Fiber-Rop/Gel**	Normal	0	100%
**Fiber-Rop/Gel-Clo**	Normal	0	100%

Video S1. Method for assessing the motor block by a four-point rating scale. The red arrow indicated the limb after sciatic nerve block.

Video S2. Method for assessing the sensory block by paw withdrawal thermal latency. The red circle indicated the limb after sciatic nerve block.

Video S3. The injectable property of the Fiber-Rop/Gel-Clo composites.

Video S4. Local anesthetic intoxication in rats. Local anesthetic intoxication was observed after perineural injection with $\geq$ 15 mg of free Rop, which manifested as limb paralysis, convulsions, respiratory depression, and eventual death. The red arrow indicated the limb after sciatic nerve block.

Video S5. Unilateral limb motor block in rats after injection with Fiber-Rop/Gel-Clo composites. The red arrow indicated the limb after sciatic nerve block.

Video S6. Normal behaviors in rats after injection with Fiber-Rop/Gel-Clo composites loaded with 20 mg of Rop. The red arrow indicated the limb after sciatic nerve block.
